# Alkyl ammonium hydrogen sulfate immobilized on Fe_3_O_4_@SiO_2_ nanoparticles: a highly efficient catalyst for the multi-component preparation of novel tetrazolo[1,5-a]pyrimidine-6-carboxamide derivatives

**DOI:** 10.1038/s41598-024-59096-2

**Published:** 2024-04-17

**Authors:** Mehdi Khalaj, Seyed Mahmoud Musavi, Majid Ghashang

**Affiliations:** 1 Department of Chemistry, Islamic Azad University, Buinzahra Branch, Buinzahra, Iran; 2 Department of Chemistry, Najafabad Branch, Islamic Azad University, Najafabad, Iran

**Keywords:** Tetrazolo[1,5-a]pyrimidine, Fe_3_O_4_@SiO_2_, Heterogeneous solid catalyst, Magnetic separation, Multi-component reaction, Environmental sciences, Chemistry, Nanoscience and technology

## Abstract

In this, a three-component reaction for the preparation of novel tetrazolo[1,5-a]pyrimidine-6-carboxamide derivatives from *N,N′*-(sulfonylbis(1,4-phenylene))bis(3-oxobutanamide), aldehydes and 1*H*-tetrazol-5-amine is reported. The application of Fe_3_O_4_@SiO_2_-(PP)(HSO_4_)_2_ (A) as a catalyst afforded the desired products **(a**_**1**_**–a**_**18**_**)** in high yields in DMF as solvent as well as under solvent-free conditions.

## Introduction

Fused poly-heterocyclic systems have long been considered essential cores in the synthesis of drugs and natural products. The wide potential applications of fused heterocycles; especially in drug discovery, have encouraged chemists to synthesize them^1^. On the other hand, any compound with a tetrazole unit is a suitable candidate for interesting pharmaceutical applications. Many compounds bearing a tetrazole moiety are known as xanthine oxidase^2^, antitubercular agents^3^, antimicrobial agents^4^, and antinociceptive active compounds^5^.

According to literature reports, fused heterocycles bearing a tetrazole core are potent compounds; especially in the field of synthetic drugs, and various methods are developed for the incorporation of tetrazoles into fused heterocycles. Some of such effective synthetic routes include C–H carbonylative annulation of *N*,1-diaryl-1*H*-tetrazol-5-amines^6^, Ugi 4-component reaction^7^, diazotization of 1-benzyloxy-5-aminotetrazoles and 1-phenethyl-5-aminotetrazoles^8^, three-component reaction of 4-chloro-3-formylcoumarins, sodium azide, alkyl/aryl acetonitriles^9^, [3 + 2]cyclization of azidotrimethylsilane with quinoxalin-2(1*H*)-ones^10^, and so-on. Additionally, there is a simple procedure comprised of the multi-component reaction of active methylene compounds such as acetoacetic esters, diverse aldehydes, and 5-amino tetrazole, which is promoted by acid/base catalysts. The targeted products, which are a series of tetrazolopyrimidines, are known for their biological potentials as analgesic materials^11^, antimicrobial and antioxidant compounds^12^, anticancer agents^13^, and antitumor materials^14^. Different reports on the synthesis of tetrazolopyrimidines using (1,2,3-triazolium-*N*-butyl sulfonic acid phosphotungstate)^15^, HMTA-BAIL@MIL-101(Cr)^16^, Fe_2_O_3_@SiO_2_-(CH_2_)_3_NHC(O)(CH_2_)_2_PPh_2_^17^, nano-Fe_3_O_4_@SiO_2_-NH-gallic acid^18^, and Mg–Al LDHs cross-linked poly triazine-urea-sulfonamide organic–inorganic hybrids have been published^19^. (MNCs) are believed to be effective alternatives for various toxic liquid acids and expensive solid catalysts. MNCs could be considered green catalysts as they can be recovered by a magnet and reused several times. Accordingly, a wide range of catalytic reactions have been reported in the literature including multi-component preparation of indeno[2′,1′:5,6]pyrido[2,3-d]pyrimidine-2,4,6(3*H*)-trione derivatives using nano Fe_2_O_3_@SiO_2_-SO_3_H^20^, synthesis of 3-(9-methyl-9*H*-carbazol-3-yl)-2-arylthiazolidin-4-one derivatives using NiFe_2_O_4_@SiO_2_ grafted alkyl sulfonic acid^21^, preparation of 14-aryl-14*H*-dibenzo[a,j]xanthene derivatives using Fe_3_O_4_@SiO_2_ functionalized sulfonic acid^22^, preparation of chromeno[4,3-d]pyrido[1,2-a]pyrimidine derivatives using NiFe_2_O_4_@SiO_2_ grafted di(3-propylsulfonic acid) nanoparticles^23^, synthesis of anticancer drugs^24^, Heck and Suzuki reactions catalyzed by palladium nanoparticles stabilized on the amino acids-functionalized Fe_3_O_4_^25^, reduction of organic pollutants by Fe_3_O_4_@CMC-Cu nano-catalyst^26^, and so-on.

Today, the main challenge in the use of catalytic systems is their ability to be recycled or not. In the absence of easy and practical recycling of catalysts, various environmental problems are created, which require a high cost to solve. Connecting functional groups such as –SO_3_H, –COOH, –NH, etc. to magnetic cores, in addition to creating heterogeneous catalytic systems, increases the possibility of easy and low-cost recycling and minimizes catalyst losses and, as a result, environmental problems. In addition, the easy recycling of the catalyst leads to a reduction in the production cost of the products. The main challenge in using magnetic particles is the very low potential of these particles in connecting to different groups and atoms. To solve this problem, magnetic particles are usually coated with polymer or silica layers, and core–shell structures like Fe_3_O_4_@SiO_2_ are created. The new structures have a high binding ability and at the same time increase the stability of the magnetic particles. Thus, the development of new magnetically separable catalysts is a great demand for synthetic chemists^27–34^.

In this study and the continuation of our research^35–44^, we intend to use a magnetic nano-catalyst for the three-component condensation of *N,N′*-(sulfonylbis(1,4-phenylene))bis(3-oxobutanamide), 1*H*-tetrazol-5-amine, and aromatic aldehydes for the synthesis of tetrazolo[1,5-a]pyrimidine-6-carboxamide derivatives. To achieve this aim, in this work Fe_3_O_4_@SiO_2_-(PP)(HSO_4_)_2_ (A) as an efficient magnetic hybrid nano-catalyst was prepared, characterized by FT-IR, XRD, FE-SEM, EDX, TGA-DTA, and VSM techniques, and was used for the catalytic synthesis of tetrazolo[1,5-a]pyrimidine-6-carboxamide derivatives.

## Materials and methods

The complete procedures, material characterization, and instruments can be found in the supplementary data file attached to this paper.

### Fe_3_O_4_ and Fe_3_O_4_@SiO_2_ nanoparticles were prepared according to our previous work^22,27^

#### General procedure

**Method 1** In a 50 mL balloon equipped with a condenser, *N,N'*-(sulfonylbis(1,4-phenylene))bis(3-oxobutanamide) (1 mmol), 1*H*-tetrazol-5-amine (2 mmol), and benzaldehyde (2 mmol), and **A** (0.025g, 0.05 mmol H^+^) were mixed in DMF (20 mL) and the mixture was mechanically stirred at 100 °C under ultrasonic irradiation for the time depicted in Table 2. After the reaction was completed (TLC following), the solvent was evaporated under reduced pressure and the solid was recrystallized from ethanol to afford the desired products.

**Method 2** a mixture of *N,N′*-(sulfonylbis(1,4-phenylene))bis(3-oxobutanamide) (1 mmol), 1*H*-tetrazol-5-amine (2 mmol), and benzaldehyde ( 2 mmol), and **A** (0.025g, 0.05 mmol H^+^) was heated at 100 °C under ultrasonic irradiation for the time depicted in Table 2. After the reaction was completed (TLC following), the was cooled and recrystallized from ethanol to afford the desired products.

### Scaleup procedure

Different experiments were performed by increasing the scale of starting materials up to 20 × and 30 ×. All experiments proceeded successfully and the desired product was achieved in high yields. (20 ×: method 1: 3.5 h, 85%, method 2: 2.9 h, 88%; 30 ×: method 1: 4 h, 87%, method 2: 3 h, 84%).

### Selected spectral data

**Scheme 1:**
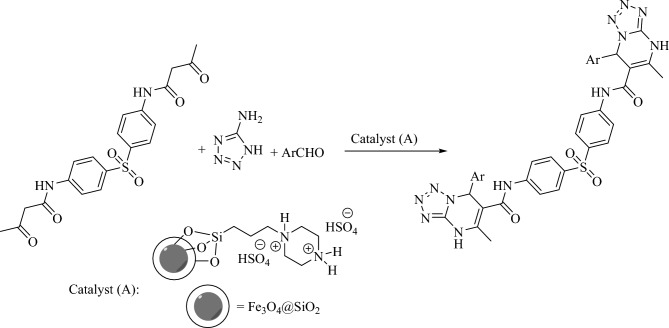
Preparation of tetrazolo[1,5-a]pyrimidine-6-carboxamide derivatives using Fe_3_O_4_@SiO_2_-(PP)(HSO_4_)_2_ (A).

***N,N'*-(Sulfonylbis(1,4-phenylene))bis(5-methyl-7-phenyl-4,7-dihydrotetrazolo[1,5-a]pyrimidine-6-carboxamide) (**Scheme 1**, Product a**_**1**_**):** ^1^H NMR (400 MHz, DMSO-*d*_*6*_): δ = 2.38 (s, 6H, CH_3_), 6.66 (s, 2H), 7.25 (t, *J* = 7.8 Hz, 4H), 7.28–33 (m, 6H), 7.38 (d, *J* = 8.0 Hz, 4H), 7.68 (d, *J* = 8.0 Hz, 4H), 8.97 (s, 2H), 10.12 (s, 2H) ppm; ^13^C NMR (100 MHz, DMSO-*d*_*6*_): δ = 19.7, 60.3, 97.7, 120.3, 124.8, 127.6, 128.1, 128.9, 130.8, 134.1, 135.7, 147.8, 151.2, 159.7 ppm; Elemental analysis: Found: C, 59.58; H, 4.23; N, 23.07; S, 4.44%; C_36_H_30_N_12_O_4_S; requires: C, 59.50; H, 4.16; N, 23.13; S, 4.41%.

***N,N′*-(Sulfonylbis(1,4-phenylene))bis(5-methyl-7-(*p*-tolyl)-4,7-dihydrotetrazolo[1,5-a]pyrimidine-6-carboxamide) (**Scheme 1**, Product a**_**2**_**):** ^1^H NMR (400 MHz, DMSO-*d*_*6*_): δ = 2.26 (s, 6H, CH_3_), 2.36 (s, 6H, CH_3_), 6.59 (s, 2H), 7.06 (d, *J* = 7.8 Hz, 4H), 7.17 (d, *J* = 7.8 Hz, 4H), 7.38 (d, *J* = 8.0 Hz, 4H), 7.66 (d, *J* = 8.0 Hz, 4H), 8.78 (s, 2H), 10.18 (s, 2H) ppm; ^13^C NMR (100 MHz, DMSO-*d*_*6*_): δ = 19.9, 21.1, 59.3, 98.6, 120.7, 124.8, 126.7, 127.9, 130.8, 134.4, 135.8, 136.9, 148.1, 151.3, 160.3 ppm; Elemental analysis: Found: C, 60.35; H, 4.49; N, 22.28; S, 4.23%; C_38_H_34_N_12_O_4_S; requires: C, 60.47; H, 4.54; N, 22.27; S, 4.25%.

***N,N'*-(Sulfonylbis(1,4-phenylene))bis(7-(4-methoxyphenyl)-5-methyl-4,7-dihydrotetrazolo[1,5-a]pyrimidine-6-carboxamide) (**Scheme 1**, Product a**_**3**_**):** ^1^H NMR (400 MHz, DMSO-*d*_*6*_): δ = 2.37 (s, 6H, CH_3_), 3.82 (s, 6H, OCH_3_), 6.47 (s, 2H), 6.94 (d, *J* = 7.8 Hz, 4H), 7.03 (d, *J* = 7.8 Hz, 4H), 7.38 (d, *J* = 8.0 Hz, 4H), 7.66 (d, *J* = 8.0 Hz, 4H), 8.78 (s, 2H), 10.18 (s, 2H) ppm; ^13^C NMR (100 MHz, DMSO-*d*_*6*_): δ = 19.8, 55.4, 59.0, 98.6, 118.7, 120.1, 123.4, 124.7, 130.4, 134.2, 135.8, 148.0, 151.4, 155.7, 160.0 ppm; Elemental analysis: Found: C, 58.09; H, 4.44; N, 21.42; S, 4.16%; C_38_H_34_N_12_O_6_S; requires: C, 58.01; H, 4.36; N, 21.36; S, 4.07%.

***N,N'*-(Sulfonylbis(1,4-phenylene))bis(7-(4-chlorophenyl)-5-methyl-4,7-dihydrotetrazolo[1,5-a]pyrimidine-6-carboxamide) (**Scheme 1**, Product a**_**4**_**):** ^1^H NMR (400 MHz, DMSO-*d*_*6*_): δ = 2.41 (s, 6H, CH_3_), 6.69 (s, 2H), 7.34–7.38 (m, 8H), 7.43 (d, *J* = 7.8 Hz, 4H), 7.67 (d, *J* = 8.2 Hz, 4H), 8.96 (s, 2H), 10.25 (s, 2H) ppm; ^13^C NMR (100 MHz, DMSO-*d*_*6*_): δ = 20.6, 62.3, 98.9, 120.4, 124.7, 128.4, 129.2, 130.6, 134.2, 136.1, 144.3, 148.2, 151.1, 160.3 ppm; Elemental analysis: Found: C, 54.38; H, 3.61; N, 21.08; S, 3.97%; C_36_H_28_Cl_2_N_12_O_4_S; requires: C, 54.34; H, 3.55; N, 21.13; S, 4.03%.

***N,N'*-(Sulfonylbis(1,4-phenylene))bis(7-(4-bromophenyl)-5-methyl-4,7-dihydrotetrazolo[1,5-a]pyrimidine-6-carboxamide) (**Scheme 1**, Product a**_**5**_**):** ^1^H NMR (400 MHz, DMSO-*d*_*6*_): δ = 2.42 (s, 6H, CH_3_), 6.72 (s, 2H), 7.38 (d, *J* = 8.0 Hz, 4H), 7.41 (d, *J* = 7.8 Hz, 4H), 7.63 (d, *J* = 7.8 Hz, 4H), 7.69 (d, *J* = 8.0 Hz, 4H), 8.91 (s, 2H), 10.22 (s, 2H) ppm; ^13^C NMR (100 MHz, DMSO-*d*_*6*_): δ = 20.5, 62.1, 98.6, 120.1, 124.6, 128.6, 129.4, 130.7, 134.8, 136.4, 146.3, 148.4, 151.0, 160.2 ppm; Elemental analysis: Found: C, 48.85; H, 3.17; N, 18.96; S, 3.64%; C_36_H_28_Br_2_N_12_O_4_S; requires: C, 48.88; H, 3.19; N, 19.00; S, 3.62%.

***N,N'*-(Sulfonylbis(1,4-phenylene))bis(5-methyl-7-(4-nitrophenyl)-4,7-dihydrotetrazolo[1,5-a]pyrimidine-6-carboxamide) (**Scheme 1**, Product a**_**6**_**):** ^1^H NMR (400 MHz, DMSO-*d*_*6*_): δ = 2.42 (s, 6H, CH_3_), 6.85 (s, 2H), 7.39 (d, *J* = 8.3 Hz, 4H), 7.68–72 (m, 8H), 8.28 (d, *J* = 7.9 Hz, 4H), 8.95 (s, 2H), 10.31 (s, 2H) ppm; ^13^C NMR (100 MHz, DMSO-*d*_*6*_): δ = 20.5, 63.6, 100.6, 120.9, 124.6, 128.7, 129.8, 130.6, 134.8, 136.7, 138.4, 148.5, 151.2, 160.5 ppm; Elemental analysis: Found: C, 52.88; H, 3.41; N, 24.04; S, 3.85%; C_36_H_28_N_14_O_8_S; requires: C, 52.94; H, 3.46; N, 24.01; S, 3.93%.

***N,N'*-(Sulfonylbis(1,4-phenylene))bis(5-methyl-7-(3-nitrophenyl)-4,7-dihydrotetrazolo[1,5-a]pyrimidine-6-carboxamide) (**Scheme 1**, Product a**_**7**_**):** ^1^H NMR (400 MHz, DMSO-*d*_*6*_): δ = 2.42 (s, 6H, CH_3_), 6.83 (s, 2H), 7.32 (t, *J* = 7.8 Hz, 2H), 7.39 (d, *J* = 8.2 Hz, 4H), 7.56 (d, *J* = 7.8 Hz, 2H), 7.70 (d, *J* = 7.9 Hz, 4H), 8.21 (d, *J* = 7.9 Hz, 2H), 8.39 (s, 2H), 8.90 (s, 2H), 10.27 (s, 2H) ppm; ^13^C NMR (100 MHz, DMSO-*d*_*6*_): δ = 20.4, 63.8, 100.1, 120.6, 124.4, 127.6, 128.1, 129.4, 130.4, 131.2, 134.8, 136.9, 138.2, 148.7, 151.0, 160.8 ppm; Elemental analysis: Found: C, 52.99; H, 3.53; N, 24.02; S, 3.81%; C_36_H_28_N_14_O_8_S; requires: C, 52.94; H, 3.46; N, 24.01; S, 3.93%.

***N,N'*-(Sulfonylbis(1,4-phenylene))bis(7-(3-chlorophenyl)-5-methyl-4,7-dihydrotetrazolo[1,5-a]pyrimidine-6-carboxamide) (**Scheme 1**, Product a**_**8**_**):** ^1^H NMR (400 MHz, DMSO-*d*_*6*_): δ = 2.39 (s, 6H, CH_3_), 6.63 (s, 2H), 7.26 (t, *J* = 7.8 Hz, 2H), 7.31 (d, *J* = 7.8 Hz, 2H), 7.38 (d, *J* = 8.2 Hz, 4H), 7.44 (d, *J* = 7.8 Hz, 2H), 7.49 (s, 2H), 7.68 (d, *J* = 7.9 Hz, 4H), 8.92 (s, 2H), 10.17 (s, 2H) ppm; ^13^C NMR (100 MHz, DMSO-*d*_*6*_): δ = 20.1, 61.3, 98.7, 120.1, 124.5, 127.2, 128.1, 129.2, 129.3, 130.4, 134.5, 136.4, 144.2, 148.0, 151.3, 160.4 ppm; Elemental analysis: Found: C, 54.41; H, 3.63; N, 21.06; S, 4.07%; C_36_H_28_Cl_2_N_12_O_4_S; requires: C, 54.34; H, 3.55; N, 21.13; S, 4.03%.

***N,N'*-(Sulfonylbis(1,4-phenylene))bis(7-(3,4-dichlorophenyl)-5-methyl-4,7-dihydrotetrazolo[1,5-a]pyrimidine-6-carboxamide) (**Scheme 1**, Product a**_**9**_**):** ^1^H NMR (400 MHz, DMSO-*d*_*6*_): δ = 2.39 (s, 6H, CH_3_), 6.66 (s, 2H), 7.33 (d, *J* = 7.8 Hz, 2H), 7.37 (d, *J* = 8.3 Hz, 4H), 7.45 (d, *J* = 7.8 Hz, 2H), 7.51 (s, 2H), 7.67 (d, *J* = 8.3 Hz, 4H), 8.88 (s, 2H), 10.14 (s, 2H) ppm; ^13^C NMR (100 MHz, DMSO-*d*_*6*_): δ = 20.3, 61.6, 98.6, 120.2, 124.6, 128.7, 129.3, 130.1, 130.5, 134.7, 136.6, 144.3, 144.8, 148.5, 151.4, 160.6 ppm; Elemental analysis: Found: C, 50.08; H, 3.09; N, 19.40; S, 3.66%; C_36_H_26_Cl_4_N_12_O_4_S; requires: C, 50.01; H, 3.03; N, 19.44; S, 3.71%.

***N,N'*-(Sulfonylbis(1,4-phenylene))bis(7-(2,4-dichlorophenyl)-5-methyl-4,7-dihydrotetrazolo[1,5-a]pyrimidine-6-carboxamide) (**Scheme 1**, Product a**_**10**_**):** ^1^H NMR (400 MHz, DMSO-*d*_*6*_): δ = 2.39 (s, 6H, CH_3_), 6.69 (s, 2H), 7.32 (d, *J* = 7.9 Hz, 2H), 7.37 (d, *J* = 8.1 Hz, 4H), 7.46 (d, *J* = 7.9 Hz, 2H), 7.53 (s, 2H), 7.68 (d, *J* = 8.1 Hz, 4H), 8.76 (s, 2H), 10.23 (s, 2H) ppm; ^13^C NMR (100 MHz, DMSO-*d*_*6*_): δ = 20.3, 61.9, 100.2, 120.6, 124.7, 128.4, 129.1, 129.5, 130.1, 134.7, 136.7, 144.1, 144.6, 148.2, 151.3, 160.4 ppm; Elemental analysis: Found: C, 49.98; H, 3.07; N, 19.43; S, 3.64%; C_36_H_26_Cl_4_N_12_O_4_S; requires: C, 50.01; H, 3.03; N, 19.44; S, 3.71%.

***N,N'*-(Sulfonylbis(1,4-phenylene))bis(7-(3,5-dichlorophenyl)-5-methyl-4,7-dihydrotetrazolo[1,5-a]pyrimidine-6-carboxamide) (**Scheme 1**, Product a**_**11**_**):** ^1^H NMR (400 MHz, DMSO-*d*_*6*_): δ = 2.39 (s, 6H, CH_3_), 6.68 (s, 2H), 7.37 (d, *J* = 8.2 Hz, 4H), 7.47 (s, 4H), 7.50 (s, 2H), 7.68 (d, *J* = 8.2 Hz, 4H), 8.85 (s, 2H), 10.19 (s, 2H) ppm; ^13^C NMR (100 MHz, DMSO-*d*_*6*_): δ = 20.0, 62.3, 99.2, 120.4, 124.5, 129.3, 130.4, 130.7, 134.7, 136.4, 144.3, 148.2, 151.1, 160.2 ppm; Elemental analysis: Found: C, 50.06; H, 3.13; N, 19.51; S, 3.78%; C_36_H_26_Cl_4_N_12_O_4_S; requires: C, 50.01; H, 3.03; N, 19.44; S, 3.71%.

***N,N'*-(Sulfonylbis(1,4-phenylene))bis(7-(2-chlorophenyl)-5-methyl-4,7-dihydrotetrazolo[1,5-a]pyrimidine-6-carboxamide) (**Scheme 1**, Product a**_**12**_**):** ^1^H NMR (400 MHz, DMSO-*d*_*6*_): δ = 2.37 (s, 6H, CH_3_), 6.61 (s, 2H), 7.25–7.28 (m, 4H), 7.32 (d, *J* = 7.8 Hz, 2H), 7.38 (d, *J* = 8.0 Hz, 4H), 7.43 (d, *J* = 7.8 Hz, 2H), 7.68 (d, *J* = 8.0 Hz, 4H), 8.90 (s, 2H), 10.19 (s, 2H) ppm; ^13^C NMR (100 MHz, DMSO-*d*_*6*_): δ = 20.1, 61.7, 98.2, 120.1, 124.4, 127.1, 127.6, 128.1, 128.4, 128.9, 134.4, 136.5, 145.2, 148.4, 151.3, 160.6 ppm; Elemental analysis: Found: C, 54.38; H, 3.67; N, 21.17; S, 4.11%; C_36_H_28_Cl_2_N_12_O_4_S; requires: C, 54.34; H, 3.55; N, 21.13; S, 4.03%.

***N,N'*-(Sulfonylbis(1,4-phenylene))bis(7-(furan-2-yl)-5-methyl-4,7-dihydrotetrazolo[1,5-a]pyrimidine-6-carboxamide) (**Scheme 1**, Product a**_**13**_**):** ^1^H NMR (400 MHz, DMSO-*d*_*6*_): δ = 2.34 (s, 6H, CH_3_), 5.81 (s, 2H), 6.49 (d, *J* = 6.8 Hz, 2H), 6.56 (t, *J* = 6.9 Hz, 2H), 7.38–7.41 (m, 6H), 7.68 (d, *J* = 8.2 Hz, 4H), 8.55 (s, 2H), 10.09 (s, 2H) ppm; ^13^C NMR (100 MHz, DMSO-*d*_*6*_): δ = 19.7, 56.7, 97.2, 104.6, 111.3, 119.7, 123.4, 124.5, 127.9, 131.4, 134.1, 148.1, 151.2, 159.7 ppm; Elemental analysis: Found: C, 54.36; H, 3.65; N, 23.71; S, 4.45%; C_32_H_26_N_12_O_6_S; requires: C, 54.39; H, 3.71; N, 23.78; S, 4.54%.

***N,N'*-(Sulfonylbis(1,4-phenylene))bis(5-methyl-7-(2-oxo-2*H*-chromen-4-yl)-4,7-dihydrotetrazolo[1,5-a]pyrimidine-6-carboxamide) (**Scheme 1**, Product a**_**14**_**):** ^1^H NMR (400 MHz, DMSO-*d*_*6*_): δ = 2.39 (s, 6H, CH_3_), 6.45 (s, 2H), 6.81 (s, 2H), 6.98 (d, *J* = 8.2 Hz, 2H), 7.38 (d, *J* = 8.1 Hz, 4H), 7.43 (t, *J* = 8.2 Hz, 2H), 7.69 (d, *J* = 8.2 Hz, 4H), 7.76 (t, *J* = 8.2 Hz, 2H), 7.92 (d, *J* = 8.1 Hz, 2H), 8.91 (s, 2H), 10.29 (s, 2H) ppm; ^13^C NMR (100 MHz, DMSO-*d*_*6*_): δ = 20.7, 63.7, 98.1, 102.3, 106.6, 119.7, 124.5, 127.8, 128.6, 129.3, 131.4, 133.7, 134.7, 138.9, 148.2, 151.2, 155.6, 161.7, 173.8 ppm; Elemental analysis: Found: C, 58.43; H, 3.55; N, 19.49; S, 3.68%; C_42_H_30_N_12_O_8_S; requires: C, 58.47; H, 3.50; N, 19.48; S, 3.72%.

**7-(9-Ethyl-9*H*-carbazol-2-yl)-*N*-(4-((4-(7-(9-ethyl-9*H*-carbazol-3-yl)-5-methyl-4,7-dihydrotetrazolo[1,5-a]pyrimidine-6-carboxamido)phenyl)sulfonyl)phenyl)-5-methyl-4,7-dihydrotetrazolo[1,5-a]pyrimidine-6-carboxamide (**Scheme 1**, Product a**_**15**_**):** ^1^H NMR (400 MHz, DMSO-*d*_*6*_): δ = 0.97 (t, *J* = 6.4 Hz, 6H), 2.37 (s, 6H, CH_3_), 3.49 (q, *J* = 6.4 Hz, 4H), 6.21 (s, 2H), 6.84 (d, *J* = 8.0 Hz, 2H), 7.02 (s, 2H), 7.18 (t, *J* = 8.0 Hz, 2H), 7.24–7.27 (m, 4H), 7.36–7.40 (m, 6H), 7.67 (d, *J* = 8.1 Hz, 4H), 7.82 (d, *J* = 8.1 Hz, 2H), 8.89 (s, 2H), 10.11 (s, 2H) ppm; ^13^C NMR (100 MHz, DMSO-*d*_*6*_): δ = 15.3, 20.8, 34.9, 64.7, 98.6, 107.1, 108.6, 111.8, 112.3, 114.9, 115.6, 123.5, 126.7, 127.4, 17.8, 128.6, 129.3, 134.4, 136.4, 137.1, 137.8, 148.2, 151.4, 162.9 ppm; Elemental analysis: Found: C, 65.07; H, 4.69; N, 20.45; S, 3.38%; C_52_H_44_N_14_O_4_S; requires: C, 64.99; H, 4.61; N, 20.40; S, 3.34%.

**5-Methyl-*N*-(4-((4-(5-methyl-7-(9-methyl-9*H*-carbazol-2-yl)-4,7-dihydrotetrazolo[1,5-a]pyrimidine-6-carboxamido)phenyl)sulfonyl)phenyl)-7-(9-methyl-9*H*-carbazol-3-yl)-4,7-dihydrotetrazolo[1,5-a]pyrimidine-6-carboxamide (**Scheme 1**, Product a**_**16**_**):** ^1^H NMR (400 MHz, DMSO-*d*_*6*_): δ = 2.37 (s, 6H, CH_3_), 3.43 (s, 6H), 6.23 (s, 2H), 6.85 (d, *J* = 8.2 Hz, 2H), 7.04 (s, 2H), 7.18 (t, *J* = 8.2 Hz, 2H), 7.23–7.27 (m, 4H), 7.35–7.39 (m, 6H), 7.67 (d, *J* = 8.0 Hz, 4H), 7.81 (d, *J* = 8.2 Hz, 2H), 8.96 (s, 2H), 10.18 (s, 2H) ppm; ^13^C NMR (100 MHz, DMSO-*d*_*6*_): δ = 20.6, 34.1, 64.7, 97.9, 107.0, 108.3, 111.2, 112.4, 114.7, 115.1, 123.9, 126.6, 127.4, 17.7, 128.6, 129.2, 134.4, 136.3, 136.8, 137.4, 148.2, 151.1, 162.2 ppm; Elemental analysis: Found: C, 64.29; H, 4.37; N, 21.06; S, 3.38%; C_50_H_40_N_14_O_4_S; requires: C, 64.37; H, 4.32; N, 21.02; S, 3.44%.

## Results and discussion

### Fe_3_O_4_@SiO_2_-(PP)(HSO_4_)_2_ (A): preparation and characterization

**Scheme 2:**
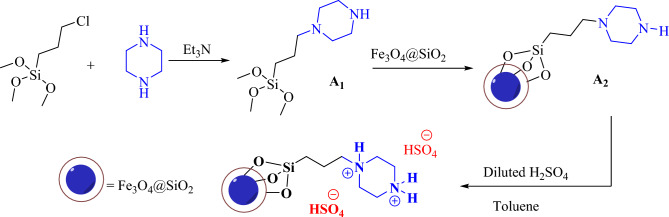
Preparation of Fe_3_O_4_@SiO_2_-(PP)(HSO_4_)_2_ (A).

**Figure 1:**
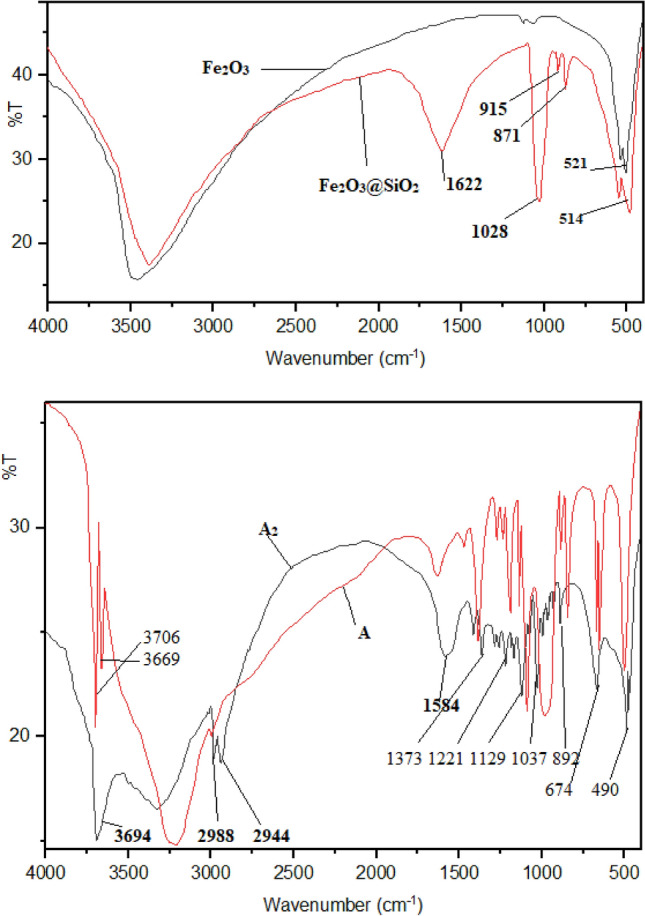
FT-IR spectra of intermediate **A**_**2**_ and Fe_3_O_4_@SiO_2_-(PP)(HSO_4_)_2_ **(A)** (Down), Fe_3_O_4_ and Fe_3_O_4_@SiO_2_ nanoparticles (Up).

First, the preparation of Fe_3_O_4_@SiO_2_-functionalized propylpiperazine-1,4-diium dihydrogensulfate (**A**) is reported. Catalyst **(A)** was prepared in three steps, as shown in Scheme 2. First, piperazine was reacted with (3-chloropropyl)trimethoxysilane to form intermediate **A**_**1**_. Et_3_N was then used to trap the HCl gas. Next, **A**_**1**_ was reacted with Fe_3_O_4_@SiO_2_ nanoparticles to form **A**_**2**_**.** The final step was the acidification of (**A**_**2**_) to form Fe_3_O_4_@SiO_2_-(PP)(HSO_4_)_2_ (**A)** as the final product. The successful grafting of the organic part to Fe_3_O_4_@SiO_2_ nanoparticles was investigated by FT-IR analysis. The FT-IR spectra of catalyst **A**, Fe_3_O_4_, intermediate **A**_**2**_, and Fe_3_O_4_@SiO_2_ nanoparticles, could be seen in Fig. 1. The FT-IR spectrum of Fe_3_O_4_ nanoparticles shows distinctive peaks below 600 cm^−1^ related to the Fe–O bonds (stretching vibration). However, in the FT-IR spectrum of Fe_3_O_4_@SiO_2_ nanoparticles, in addition to the peak corresponding to the F-O bond (below 600 cm^−1^), there are distinctive peaks as Si–O, Si–OH, and Si–O–Si at 1622, 1028, 915, and 871 cm^−1^ (stretching and bending vibrations) respectively. The FT-IR spectrum of intermediate **A**_**2**_ shows distinctive peaks at 3694 (N–H), 2988, 2944 (C–H), 1584 (Si–O), 1373, 1221, 1129, 1037, 892, 674, and 490 (Fe–O) cm^−1^ ascribed to the vibration of C–H, C–C, Si–O-Si, Fe–O and C–N bonds. Compared to the FT-IR spectra of A_2_, some changes are observed in the spectra of sample **A**. The peaks located at 3706 and 3669cm^−1^ are related to the N–H vibration bonds. The sulfonic acid groups have a broad peak at 3000–3600 cm^−1^.

**Figure 2:**
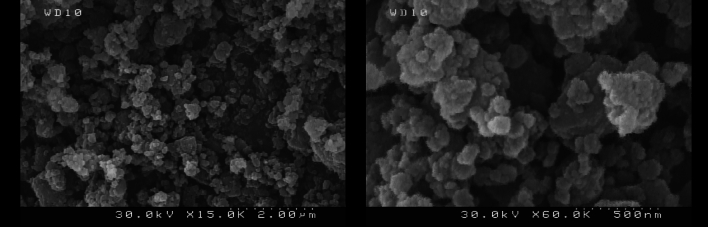
FE-SEM images of Fe_3_O_4_@SiO_2_-(PP)(HSO_4_)_2_ **(A).**

FE-SEM images (Fig. 2) were used to investigate the surface morphology of the as-prepared Fe_3_O_4_@SiO_2_-(PP)(HSO_4_)_2_ **(A**). As observed in the images, the sample has a homogeneously spherical morphology with an average diameter of less than 100 nm.

**Figure 3:**
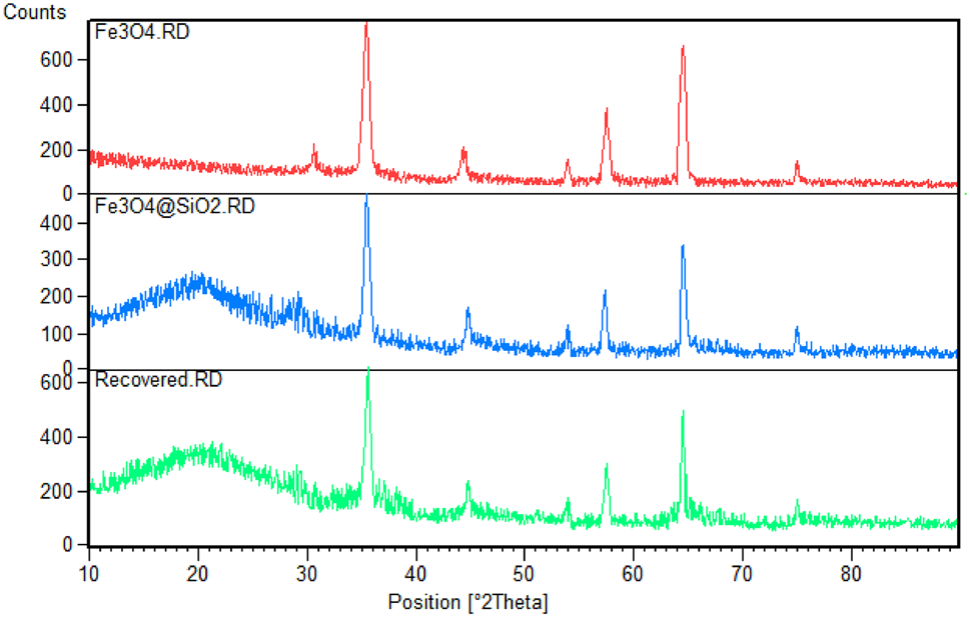
XRD patterns of Fe_3_O_4_, fresh, and recovered Fe_3_O_4_@SiO_2_-(PP)(HSO_4_)_2_ **(A)** catalyst.

To further characterize Fe_3_O_4_@SiO_2_-(PP)(HSO_4_)_2_ (A), the samples were subjected to XRD analysis to determine the crystalline phases. Figure 3 shows the XRD patterns of Fe_3_O_4_ and (**A**). The XRD pattern of Fe_3_O_4_ nanoparticles demonstrates prominent peaks at 30.6, 35.4, 44.3, 53.9, 57.4, 64.5, and 75.0 [2Ɵ°], indicating a cubic structure for Fe_3_O_4_ [Reference code: 00-001-1111]. The XRD pattern of sample **(A)** shows a similar pattern with a shoulder located in the 10–30 (2Ɵ°) range, which may be due to the amorphous phase of silica. Furthermore, the peaks related to the Fe_3_O_4_ phase have lower intensities due to the integration of organic parts and the SiO_2_ phase.

### Fe_3_O_4_@SiO_2_-(PP)(HSO_4_)_2_ (A): thermal stability and chemical composition

**Figure 4:**
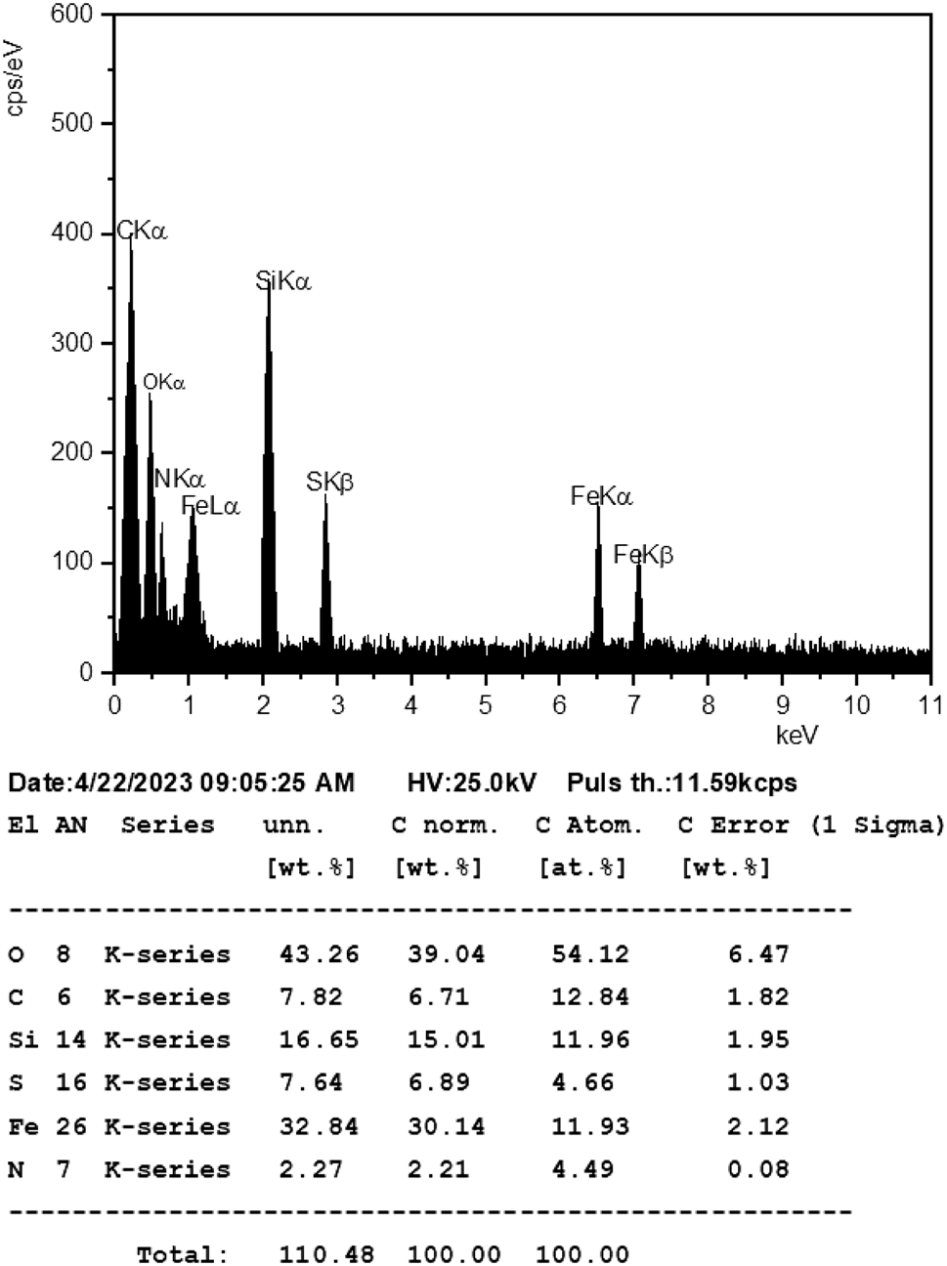
EDX analysis of Fe_3_O_4_@SiO_2_-(PP)(HSO_4_)_2_ **(A).**

The chemical composition of Fe_3_O_4_@SiO_2_-(PP)(HSO_4_)_2_ **(A)** was determined by EDX analysis (Fig. 4). The EDX analysis indicates the presence of Fe (32.84%), Si (15.01%), S (6.89%), N (2.21%), C (6.71%), and O (39.04%), confirming the integration of the organic part and sulfate group into Fe_3_O_4_@SiO_2_. The presence of Fe, Si, S, C, N, and O elements indicates the formation of Fe_3_O_4_@SiO_2_-(PP)(HSO_4_)_2_ **(A)**.

**Figure 5:**
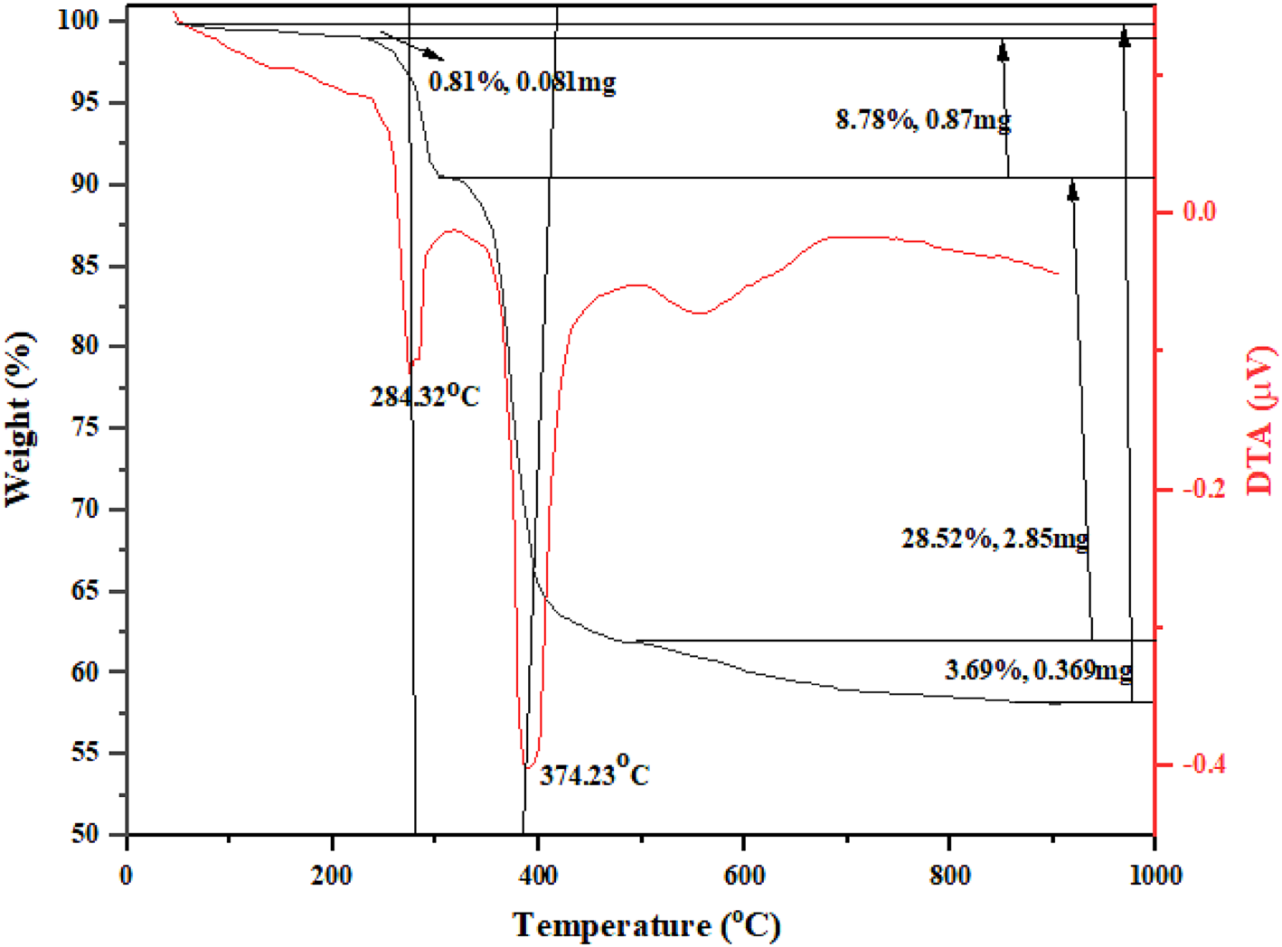
TGA-DTA analysis of Fe_3_O_4_@SiO_2_-(PP)(HSO_4_)_2_ **(A).**

Next, the thermal behavior of (**A**) was investigated by TGA-DTA analysis (Fig. 5). The sample is stable up to 200 °C and shows four different mass losses due to the removal of the adsorbed water (50–200 °C), removal of SO_X_ gases (200–320 °C), decomposition of the organic part by the removal of CO_2_, H_2_O, and NOx gases (300–550 °C), and the formation of SiO_2_ phase (500–800 °C).^45^ Accordingly, the ratio of inorganic to organic parts is nearly 2/1, which is close to the ratio of the initial substrates.

### Determination of active sites

The sample has an acidic nature and thus, the determination of H^+^ values is important to investigate the role and determine the conditions for the application of the sample as a catalyst. The values of H^+^ were determined by EDX analysis, TGA method, and barium sulfate (BaSO_4_) titration-precipitation test. The obtained results are shown in Table 1.

**Table 1:** Determination of H^+^ values of Fe_3_O_4_@SiO_2_-(PP)(HSO_4_)_2_ **(A)**.

EDX analysis	TGA method	BaSO_4_ test
2.15 mmolg^−1^	2.71 mmolg^−1^	2.03 mmolg^−1^

The sulfur values of the sample were determined through the sulfur element percent in the results of EDX analysis (S, 6.89%). Similarly, the amount of S atoms could be determined by the values of SO_x_ removal using TGA. The BaSO_4_ method involves titration by barium chloride solution. Accordingly, the H^+^ capacities of the sample were found to be 2.15, 2.71, and 2.03 mmol H^+^/g by EDX, TGA, and BaSO_4_ tests, respectively.

**Figure 6:**
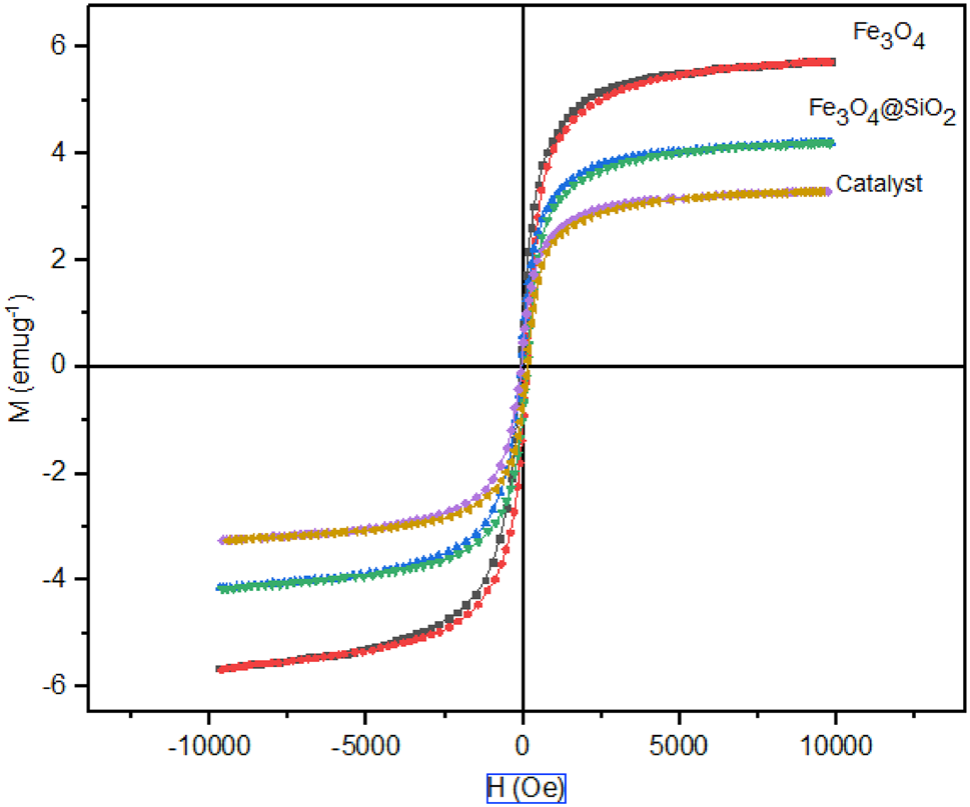
VSM analysis of **(A)**, Fe_3_O_4_, and Fe_3_O_4_@SiO_2_ samples.

To assure the desirable performance and facile separation of the nano-catalyst, by a magnetic field, VSM analysis was used. Figure 6 shows the plotted results of VSM analysis performed at 25°C within the magnetic field of − 10,000 to 10,000 Oe. According to the hysteresis curves shown in Fig. 6, the functionalization of the Fe_3_O_4_ decreased the VSM characteristic values including saturation magnetization (M_s_), remanence magnetization (M_r_), and coercivity field (H_c_) (Table 2). However, Fe_3_O_4_ exhibited a considerable magnetic nature.

**Table 2:** Magnetic parameters of Fe_3_O_4_, Fe_3_O_4_@SiO_2_, and A.

Sample	M_s_ (memu/g)	M_r_ (memu/g)	H_c_ (Oe)
Fe_3_O_4_	5.71	0.919	− 71.45
Fe_3_O_4_@SiO_2_	4.19	0.604	− 63.99
A	3.28	0.474	− 61.88

In organic–inorganic hybrids, such as those used here, the organic part hurts the saturation magnetization. The organic chain has a diamagnetic effect and accordingly, the sample **(A)** shows lower magnetic saturation than Fe_3_O_4_ and Fe_3_O_4_@SiO_2_ samples. In addition, our observations confirm the easy recovery of the catalyst by an external magnet.

### Preparation of tetrazolo[1,5-a]pyrimidine-6-carboxamide derivatives

#### Reaction condition optimization

The prepared sample **(A)** was then used in the synthesis of tetrazolo[1,5-a]pyrimidine-6-carboxamide derivatives to act as a catalyst. Initially, the reaction of *N,N'*-(sulfonylbis(1,4-phenylene))bis(3-oxobutanamide), 1*H*-tetrazol-5-amine, and benzaldehyde was chosen as a model for the synthesis of 5-methyl-*N*-(4-((4-(5-methyl-7-phenyl-4,5,6,7-tetrahydrotetrazolo[1,5-a]pyrimidine-6-carboxamido)phenyl)sulfonyl)phenyl)-7-phenyl-4,7-dihydrotetrazolo[1,5-a]pyrimidine-6-carboxamide **(a**_**1**_**)**. To determine the optimal conditions in the synthesis of compound **(a**_**1**_**)**, the model reaction was studied using different solvents, catalyst dosages, and temperatures (Table 3). According to the results obtained, the reaction did not proceed at low temperatures. In addition, non-polar, less polar, and polar solvents with boiling point less than 100°C such as hexane, dichloromethane (CH_2_Cl_2_), chloroform (CHCl_3_), and ethyl acetate (EtOAc) were not suitable for the reaction. In aqueous media, no products were formed. Upon increasing the reaction temperature up to 100 °C, the reaction yields in tetrahydrofuran (THF) and toluene were 56 and 20%, respectively. Notably, the reaction had an acceptable yield in dimethyl formamide (DMF, 79%). To obtain better product yields, ultrasonic irradiation (US) was used. A high product yield (95%) was obtained under ultrasonic irradiation using DMF solvent. Notably, under solvent-free conditions and ultrasonic irradiation, the desired product (a_1_) was formed in a high yield (91%) at 100 °C.

**Table 3:** Optimization of the reaction conditions.

Entry	Catalyst	Condition	Time (h)	Yield (%)*
1	0.05g, 0.1 mmol H^+^	EtOH, Reflux	3	24
2	0.05g, 0.1 mmol H^+^	EtOH, r.t	3	–
3	0.05g, 0.1 mmol H^+^	THF, 100 °C	3	56
4	0.05g, 0.1 mmol H^+^	Hexane, Reflux	3	–
5	0.05g, 0.1 mmol H^+^	CH_2_Cl_2_, Reflux	3	–
6	0.05g, 0.1 mmol H^+^	CHCl_3_, Reflux	3	–
7	0.05g, 0.1 mmol H^+^	Toluene, Reflux	3	20
8	0.05g, 0.1 mmol H^+^	DMF, 100 °C	3	79
9	0.05g, 0.1 mmol H^+^	H_2_O, Reflux	4	–
10	0.05g, 0.1 mmol H^+^	EtOAc, Reflux	3	–
11	0.05g, 0.1 mmol H^+^	DMF, 100 °C, Ultrasonic Irradiation	3	95
12	0.05g, 0.1 mmol H^+^	Solvent-free, 100 °C, Ultrasonic Irradiation	2	91
13	0.01g, 0.02 mmol H^+^	DMF, 100 °C, Ultrasonic Irradiation	4	47
14	0.025g, 0.05 mmol H^+^	DMF, 100 °C, Ultrasonic Irradiation	3	92
15	0.075g, 0.15 mmol H^+^	DMF, 100 °C, Ultrasonic Irradiation	3	91
16	0.1g, 0.2 mmol H^+^	DMF, 100 °C, Ultrasonic Irradiation	3	89
17	–	DMF, 100 °C, Ultrasonic Irradiation	5	–

*Isolated Yield; based on the preparation of **a**_**1**_.

Next, the reaction was investigated using different dosages of the catalyst. In the absence of the catalyst, no product was formed. The results revealed that 0.5 g of the catalyst gave the highest yield of the product (**a**_**1**_)**.** Thus, DMF solvent and solvent-free conditions were selected as the two best media for the reaction while the suitable catalyst dosage was determined as 0.05 g, as it provided the highest yields at reasonable reaction times (Table 3).

Under the optimized conditions, the scope of the reaction was expanded using various aromatic and aliphatic aldehydes. The results are shown in Table 3. Accordingly, when aliphatic aldehydes were used, no product was formed. However, different aromatic aldehydes were found to be appropriate substrates in the reaction. The electronic effects of the substituents on the aromatic ring in the aromatic aldehydes are expected to affect the reaction rate. Based on the results obtained, electron-donating substituents increased the reaction rate, contrary to electron-withdrawing groups (Table 4).

**Table 4:** Preparation of **(a**_**1**_–**a**_**18**_**).**

Aldehyde	Product	Method 1: Time (h)/Yield (%)*	Method 2: Time (h)/Yield (%)*	M.p. (°C)
	**a**_**1**_	3/95	2/91	289–291
	**a**_**2**_	1.5/86	1.5/89	276–278
	**a**_**3**_	1.5/85	1.5/93	279–281
	**a**_**4**_	4/95	3/96	˃300
	**a**_**5**_	4/96	3/90	˃300
	**a**_**6**_	5/89	4/85	˃300
	**a**_**7**_	5/92	4/96	˃300
	**a**_**8**_	4/95	3.5/94	296–298
	**a**_**9**_	2.5/96	2/93	˃300
	**a**_**10**_	2/94	1.5/92	˃300
	**a**_**11**_	2/97	1.5/95	˃300
	**a**_**12**_	4/95	3/91	293–295
	**a**_**13**_	2.5/82	2/85	266–268
	**a**_**14**_	3/93	2.5/89	˃300
	**a**_**15**_	3/94	2.592	˃300
	**a**_**16**_	3/95	2.5/98	˃300
	**a**_**17**_	2.5/–	2/–	–
	**a**_**18**_	3/–	2/–	–

*Isolated Yields; Method 1: DMF, 100 °C, Ultrasonic Irradiation; Method 2: Solvent-free, 100 °C, Ultrasonic Irradiation.

**Scheme 3:**
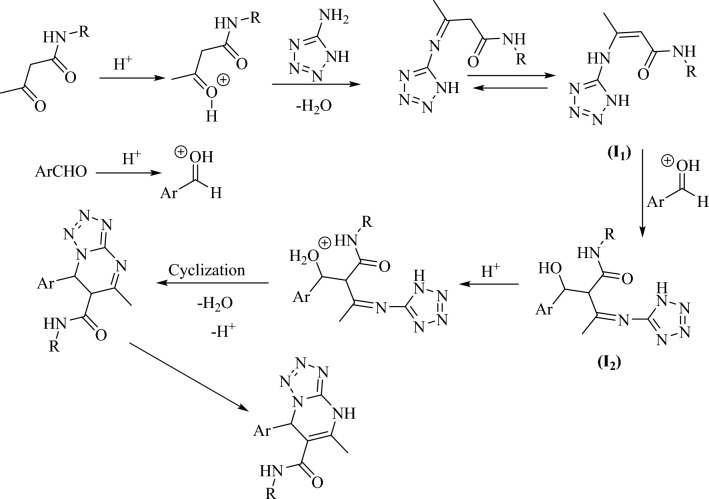
Proposed mechanism for the synthesis of tetrazolo[1,5-a]pyrimidine-6-carboxamide derivatives using Fe_3_O_4_@SiO_2_-(PP)(HSO_4_)_2_ (**A**).

Scheme 3 shows a plausible proposed reaction mechanism for the synthesis of compounds **a**_**1**_**–a**_**18**_. As suggested, the Brønsted acid catalyst activates the carbonyl groups. The reaction starts with the reaction of NH_2_ group with the activated carbonyl groups to form an enamine active compound (Intermediate **I**_**1**_). The next step is the reaction of **I**_**1**_ with the activated aldehyde to form **I**_**2**_. Intermediate **I**_**2**_ undergoes cyclization and enamine formation to yield the final products.

**Figure 7:**
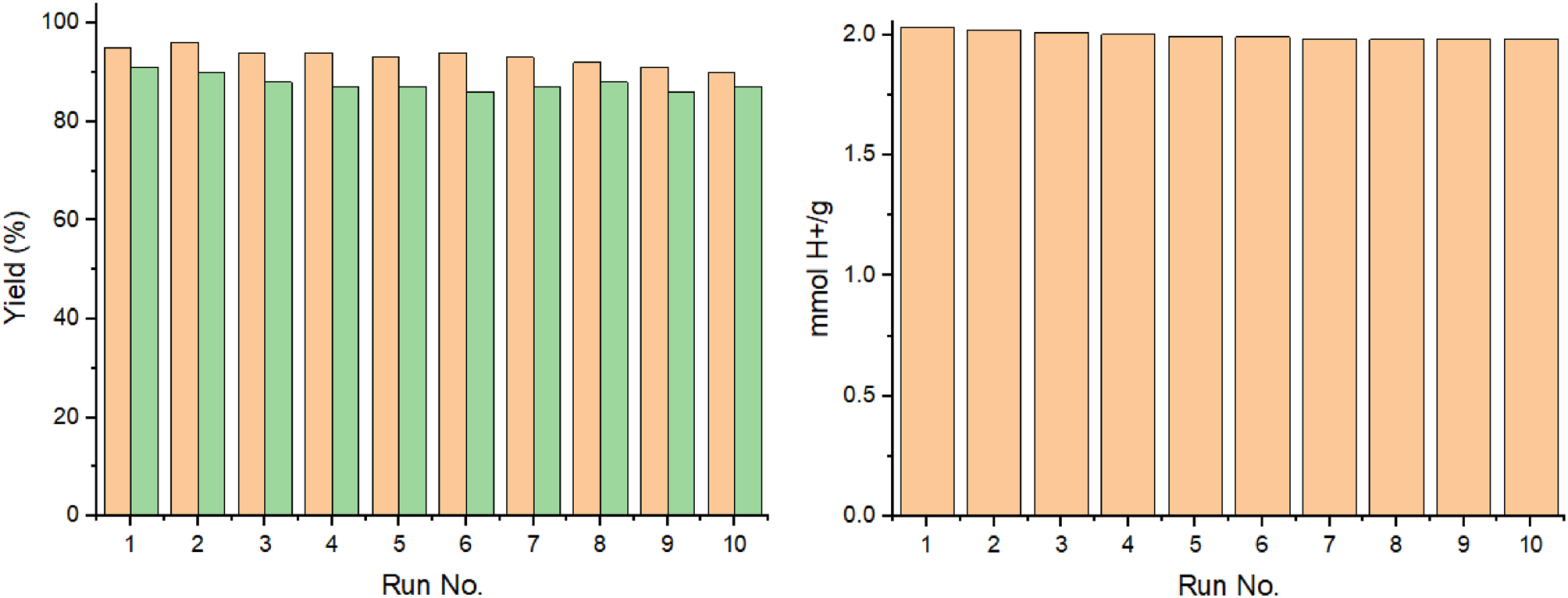
Recovery of **A** (Methods 1,2) and leaching test results in the synthesis of (**a**_**1**_).

Finally, an external magnet could be used to recover the catalyst, which was then washed with ethanol, dried, and used again. The preparation of (**a**_**1**_) was chosen for the recovery test. The recovery experiments showed acceptable results after 10 catalytic runs (Fig. 7). The XRD pattern of the recovered catalyst confirmed the stability of the catalyst during the reaction (Fig. 1). In addition, after each run, the recovered catalyst was tested using titration by barium chloride solution. The results indicated good catalyst stability and no clear leaching was observed.

## Conclusion

In this work, tetrazolo[1,5-a]pyrimidine-6-carboxamide derivatives were prepared using Fe_3_O_4_@SiO_2_-(PP)(HSO_4_)_2_ (**A**) as a catalyst. The TGA-DTA analysis indicated the stability of this organic–inorganic hybrid up to 200 °C. In addition, the ratio of the inorganic to organic parts was 2/1, which was close to that of the initial substrates. Using the barium chloride titration test, the H^+^ capacity of the sample was determined to be 2.03 mmol H^+^/g. The XRD pattern of the fresh and recovered samples (**A**) confirmed the stability of the catalyst. The results showed promising potential and easy recovery of magnetic nano-catalysts. The obtaining of reasonably high yields in short reaction times and readily available starting materials make this protocol potentially useful in organic synthesis.

### Supplementary Information

Supplementary Information.

## Data Availability

The spectral data, which could support our findings, are available as a supplementary material attached to this article.
