# Supplementary material for: Alkyl ammonium hydrogen sulfate immobilized on Fe3O4@SiO2 nanoparticles: a highly efficient catalyst for the multi-component preparation of novel tetrazolo[1,5-a]pyrimidine-6-carboxamide derivatives
Source: Sci Rep. 2024 Apr 17;14:8870. doi: 10.1038/s41598-024-59096-2 (PMC11024118; doi:10.1038/s41598-024-59096-2)
Supplement: Supplementary file 1 — Supplementary Information. [file 41598_2024_59096_MOESM1_ESM.docx]

**Alkyl ammonium hydrogen sulfate immobilized on Fe_3_O_4_@SiO_2_ nanoparticles: a highly efficient catalyst for the multi-component preparation of novel tetrazolo[1,5-a]pyrimidine-6-carboxamide derivatives**

Mehdi Khalaj^^[[1]](#footnote-1)^*a^ Seyed Mahmoud Musavi^a^ and Majid Ghashang^b^

^a^Department of Chemistry, Islamic Azad University, Buinzahra Branch, Buinzahra, Iran

^b^Department of Chemistry, Najafabad Branch, Islamic Azad University, Najafabad, Iran

# 2. Materials and Methods

Chemicals including, FeCl_3_.6H_2_O (97%; CAS-Number: 10025-77-1), Si(OEt)_4_ (˃99.9%; CAS-Nummer:78-10-4), FeCl_2_.4H_2_O (98%; CAS-Nummer: 13478-10-9), and organic materials (in pure form and synthetic grade) were purchased from the commercial suppliers of Merck and Aldrich companies products. X-ray diffraction (XRD) analysis was performed by a Philips X-Ray diffractometer (Model: D5000). A Hitachi microscope (model: S-4160) was used for the morphology investigations through the analysis of FE-SEM images. The thermal stability analysis of the catalyst was done by a TA (Q600) instrument through the TGA-DTA tests. The Thermo (AVATAR) instrument was used for the solid phase FT-IR analysis which was recorded on a KBr disk. The NMR spectra were recorded and measured in CDCl_3_ as solvent on a Bruker Avance DPX 400 MHz instrument.

**Preparation of Fe_3_O_4_ nanoparticles**

In a 500 mL beaker, 50 mmol of each salt of FeCl_3_.6H_2_O and FeCl_2_.4H_2_O were dissolved in 200 mL deionized water. Subsequently, an aqueous solution of ammonia (15%) was added to the solution dropwise until the pH value was reached 11. The black precipitate was separated by an external magnet and washed with water twice and dried in air.

**Preparation of Fe_3_O_4_@SiO_2_ nanoparticles**

Magnetic Fe_3_O_4_ nanoparticles were coated with silica using the following procedure. Typically, Fe_3_O_4_ nanoparticles (3g) were dispersed into an aqueous ethanol solution (80%) and the pH of the solution was adjusted to 9 with the dropwise addition of ammonia solution (25%). Subsequently, 7 mL tetraethyl orthosilicate (TEOS) was dissolved in 20 mL ethanol and dropwise added to the dispersed Fe_3_O_4_ solution under mechanical stirring. The mixture was stirred for 48h and was separated by an external magnet and dried in air at 80°C.

**Preparation of Fe_3_O_4_@SiO_2_-(PP)(HSO_4_)_2_ (A) :**

**Stage 1:**

In a 250 mL two-necked round-bottomed flask equipped with a condenser, piperazine (6 mmol) and 5 mL of triethylamine were dissolved in 50 mL of toluene and the mixture was stirred for 30 min at 25°C. Subsequently, after the addition of (3-Chloropropyl)trimethoxysilane (6 mmol), the mixture was refluxed overnight to complete the reaction (Scheme 2, **A_1_**). Next, the mixture was cooled to room temperature (25°C) and combined with Fe_3_O_4_@SiO_2_ nanoparticles (3 g) under stirring. The mixture was again refluxed for 5h. Afterward, the solid was separated, washed with toluene, and dried at 80 °C (Scheme 2, **A_2_**).

Intermediate A_1_ could be purified by recrystallization of the sample in CH_2_Cl_2_. The sample was kept in desiccator under vacuum before analysis.

**Product A_1_:** ^1^H NMR (400 MHz, DMSO-*d_6_*): δ = 0.49 (t, *J* = 6.8 Hz, 2H, CH_2_Si), 1.02 (s, 1H, NH), 1.13-1.17 (m, 2H), 2.88-2.93 (m, 10H), 3.36 (s, 9H, OCH_3_) ppm


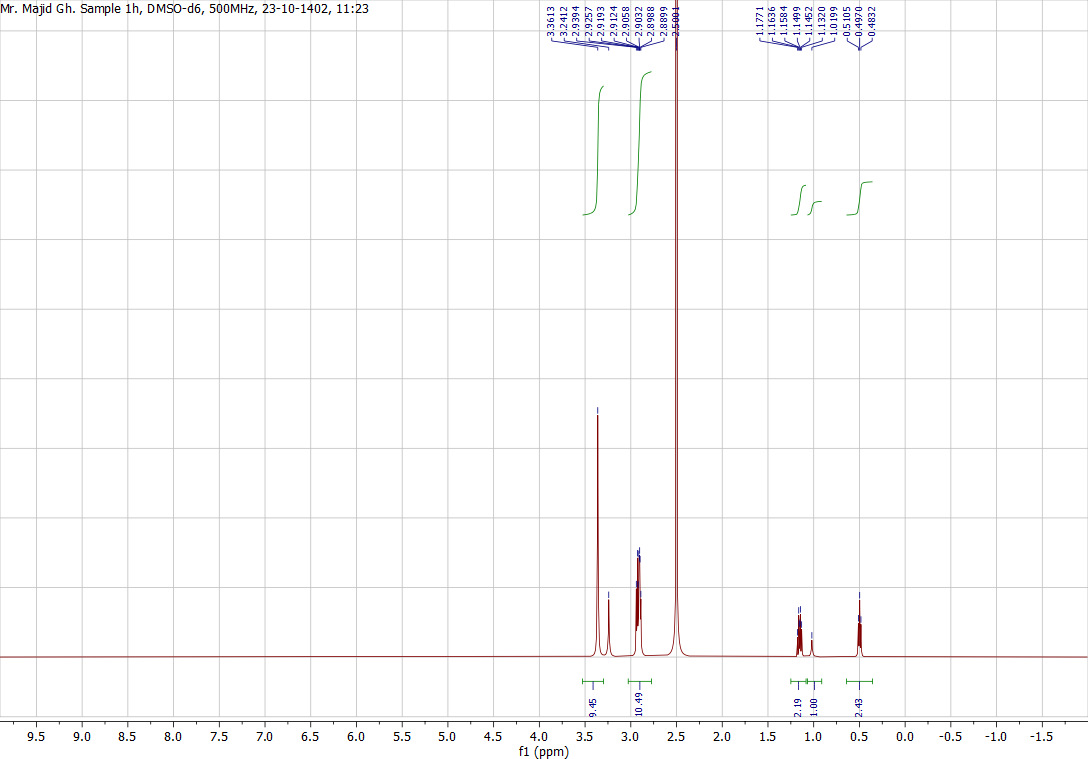


**Figure A_1_:** ^1^H NMR specrtum of intermediate A_1_

**Stage 2:**

In a 250 mL two-necked round-bottomed flask equipped with a condenser, product **A_2_** (5g) was dispersed in 100 mL of toluene, combined dropwise with diluted sulfuric acid (15 mL), and the mixture was stirred for 4h. Then, the solvent was evaporated and the solid was washed with acetone, and dried.

**Acidity measurement using Barium sulfate test**

1 g of the as prepared catalyst was dispersed in 100 mL of deionized water and combined with a solution of barium chloride (1 M). the sample was aged for BaSO_4_ precipitation. The collected solid was carefully weighed and used to determine the amount of SO_4_^2-^ ions. The SO_4_^2-^ values of the catalyst was determined as 2.03 mmol/g. Accordingly, the H^+^ capacity of the sample was determined to be 2.03 mmol H^+^/g.

**General procedure:**

**Method 1:** In a 50 mL balloon equipped with a condenser, *N,N'*-(sulfonylbis(1,4-phenylene))bis(3-oxobutanamide) (1 mmol), 1*H*-tetrazol-5-amine (2 mmol), and benzaldehyde ( 2 mmol), and **A** (0.025g, 0.05 mmol H^+^) were mixed in DMF (20 mL) and the mixture was mechanically stirred at 100°C under ultrasonic irradiation fot the time depicted in Table 2. After of the reaction was completed (TLC following), the solvent was evaporated under reduced pressure and the solid was recrystallized from ethanol to afford the desired products.

**Method 2:** a mixture of *N,N'*-(sulfonylbis(1,4-phenylene))bis(3-oxobutanamide) (1 mmol), 1*H*-tetrazol-5-amine (2 mmol), and benzaldehyde ( 2 mmol), and **A** (0.025g, 0.05 mmol H^+^) was heated at 100°C under ultrasonic irradiation fot the time depicted in Table 2. After of the reaction was completed (TLC following), the was cooled and recrystallized from ethanol to afford the desired products.

**Selected spectral data:**

***N,N'*-(sulfonylbis(1,4-phenylene))bis(5-methyl-7-phenyl-4,7-dihydrotetrazolo[1,5-a]pyrimidine-6-carboxamide) (Scheme 1, Product a_1_):** ^1^H NMR (400 MHz, DMSO-*d_6_*): δ = 2.38 (s, 6H, CH_3_), 6.66 (s, 2H), 7.25 (t, *J* = 7.8 Hz, 4H), 7.28-33 (m, 6H), 7.38 (d, *J* = 8.0 Hz, 4H), 7.68 (d, *J* = 8.0 Hz, 4H), 8.97 (s, 2H), 10.12 (s, 2H) ppm; ^13^C NMR (100 MHz, DMSO-*d_6_*): δ = 19.7, 60.3, 97.7, 120.3, 124.8, 127.6, 128.1, 128.9, 130.8, 134.1, 135.7, 147.8, 151.2, 159.7 ppm; Elemental analysis: Found: C, 59.58; H, 4.23; N, 23.07; S, 4.44%; C_36_H_30_N_12_O_4_S; requires: C, 59.50; H, 4.16; N, 23.13; S, 4.41%.

***N,N'*-(sulfonylbis(1,4-phenylene))bis(5-methyl-7-(*p*-tolyl)-4,7-dihydrotetrazolo[1,5-a]pyrimidine-6-carboxamide) (Scheme 1, Product a_2_):** ^1^H NMR (400 MHz, DMSO-*d_6_*): δ = 2.26 (s, 6H, CH_3_), 2.36 (s, 6H, CH_3_), 6.59 (s, 2H), 7.06 (d, *J* = 7.8 Hz, 4H), 7.17 (d, *J* = 7.8 Hz, 4H), 7.38 (d, *J* = 8.0 Hz, 4H), 7.66 (d, *J* = 8.0 Hz, 4H), 8.78 (s, 2H), 10.18 (s, 2H) ppm; ^13^C NMR (100 MHz, DMSO-*d_6_*): δ = 19.9, 21.1, 59.3, 98.6, 120.7, 124.8, 126.7, 127.9, 130.8, 134.4, 135.8, 136.9, 148.1, 151.3, 160.3 ppm; Elemental analysis: Found: C, 60.35; H, 4.49; N, 22.28; S, 4.23%; C_38_H_34_N_12_O_4_S; requires: C, 60.47; H, 4.54; N, 22.27; S, 4.25%.

***N,N'*-(sulfonylbis(1,4-phenylene))bis(7-(4-methoxyphenyl)-5-methyl-4,7-dihydrotetrazolo[1,5-a]pyrimidine-6-carboxamide) (Scheme 1, Product a_3_):** ^1^H NMR (400 MHz, DMSO-*d_6_*): δ = 2.37 (s, 6H, CH_3_), 3.82 (s, 6H, OCH_3_), 6.47 (s, 2H), 6.94 (d, *J* = 7.8 Hz, 4H), 7.03 (d, *J* = 7.8 Hz, 4H), 7.38 (d, *J* = 8.0 Hz, 4H), 7.66 (d, *J* = 8.0 Hz, 4H), 8.78 (s, 2H), 10.18 (s, 2H) ppm; ^13^C NMR (100 MHz, DMSO-*d_6_*): δ = 19.8, 55.4, 59.0, 98.6, 118.7, 120.1, 123.4, 124.7, 130.4, 134.2, 135.8, 148.0, 151.4, 155.7, 160.0 ppm; Elemental analysis: Found: C, 58.09; H, 4.44; N, 21.42; S, 4.16%; C_38_H_34_N_12_O_6_S; requires: C, 58.01; H, 4.36; N, 21.36; S, 4.07%.

***N,N'*-(sulfonylbis(1,4-phenylene))bis(7-(4-chlorophenyl)-5-methyl-4,7-dihydrotetrazolo[1,5-a]pyrimidine-6-carboxamide) (Scheme 1, Product a_4_):** ^1^H NMR (400 MHz, DMSO-*d_6_*): δ = 2.41 (s, 6H, CH_3_), 6.69 (s, 2H), 7.34-7.38 (m, 8H), 7.43 (d, *J* = 7.8 Hz, 4H), 7.67 (d, *J* = 8.2 Hz, 4H), 8.96 (s, 2H), 10.25 (s, 2H) ppm; ^13^C NMR (100 MHz, DMSO-*d_6_*): δ = 20.6, 62.3, 98.9, 120.4, 124.7, 128.4, 129.2, 130.6, 134.2, 136.1, 144.3, 148.2, 151.1, 160.3 ppm; Elemental analysis: Found: C, 54.38; H, 3.61; N, 21.08; S, 3.97%; C_36_H_28_Cl_2_N_12_O_4_S; requires: C, 54.34; H, 3.55; N, 21.13; S, 4.03%.

***N,N'*-(sulfonylbis(1,4-phenylene))bis(7-(4-bromophenyl)-5-methyl-4,7-dihydrotetrazolo[1,5-a]pyrimidine-6-carboxamide) (Scheme 1, Product a_5_):** ^1^H NMR (400 MHz, DMSO-*d_6_*): δ = 2.42 (s, 6H, CH_3_), 6.72 (s, 2H), 7.38 (d, *J* = 8.0 Hz, 4H), 7.41 (d, *J* = 7.8 Hz, 4H), 7.63 (d, *J* = 7.8 Hz, 4H), 7.69 (d, *J* = 8.0 Hz, 4H), 8.91 (s, 2H), 10.22 (s, 2H) ppm; ^13^C NMR (100 MHz, DMSO-*d_6_*): δ = 20.5, 62.1, 98.6, 120.1, 124.6, 128.6, 129.4, 130.7, 134.8, 136.4, 146.3, 148.4, 151.0, 160.2 ppm; Elemental analysis: Found: C, 48.85; H, 3.17; N, 18.96; S, 3.64%; C_36_H_28_Br_2_N_12_O_4_S; requires: C, 48.88; H, 3.19; N, 19.00; S, 3.62%.

***N,N'*-(sulfonylbis(1,4-phenylene))bis(5-methyl-7-(4-nitrophenyl)-4,7-dihydrotetrazolo[1,5-a]pyrimidine-6-carboxamide) (Scheme 1, Product a_6_):** ^1^H NMR (400 MHz, DMSO-*d_6_*): δ = 2.42 (s, 6H, CH_3_), 6.85 (s, 2H), 7.39 (d, *J* = 8.3 Hz, 4H), 7.68-72 (m, 8H), 8.28 (d, *J* = 7.9 Hz, 4H), 8.95 (s, 2H), 10.31 (s, 2H) ppm; ^13^C NMR (100 MHz, DMSO-*d_6_*): δ = 20.5, 63.6, 100.6, 120.9, 124.6, 128.7, 129.8, 130.6, 134.8, 136.7, 138.4, 148.5, 151.2, 160.5 ppm; Elemental analysis: Found: C, 52.88; H, 3.41; N, 24.04; S, 3.85%; C_36_H_28_N_14_O_8_S; requires: C, 52.94; H, 3.46; N, 24.01; S, 3.93%.

***N,N'*-(sulfonylbis(1,4-phenylene))bis(5-methyl-7-(3-nitrophenyl)-4,7-dihydrotetrazolo[1,5-a]pyrimidine-6-carboxamide) (Scheme 1, Product a_7_):** ^1^H NMR (400 MHz, DMSO-*d_6_*): δ = 2.42 (s, 6H, CH_3_), 6.83 (s, 2H), 7.32 (t, *J* = 7.8 Hz, 2H), 7.39 (d, *J* = 8.2 Hz, 4H), 7.56 (d, *J* = 7.8 Hz, 2H), 7.70 (d, *J* = 7.9 Hz, 4H), 8.21 (d, *J* = 7.9 Hz, 2H), 8.39 (s, 2H), 8.90 (s, 2H), 10.27 (s, 2H) ppm; ^13^C NMR (100 MHz, DMSO-*d_6_*): δ = 20.4, 63.8, 100.1, 120.6, 124.4, 127.6, 128.1, 129.4, 130.4, 131.2, 134.8, 136.9, 138.2, 148.7, 151.0, 160.8 ppm; Elemental analysis: Found: C, 52.99; H, 3.53; N, 24.02; S, 3.81%; C_36_H_28_N_14_O_8_S; requires: C, 52.94; H, 3.46; N, 24.01; S, 3.93%.

***N,N'*-(sulfonylbis(1,4-phenylene))bis(7-(3-chlorophenyl)-5-methyl-4,7-dihydrotetrazolo[1,5-a]pyrimidine-6-carboxamide) (Scheme 1, Product a_8_):** ^1^H NMR (400 MHz, DMSO-*d_6_*): δ = 2.39 (s, 6H, CH_3_), 6.63 (s, 2H), 7.26 (t, *J* = 7.8 Hz, 2H), 7.31 (d, *J* = 7.8 Hz, 2H), 7.38 (d, *J* = 8.2 Hz, 4H), 7.44 (d, *J* = 7.8 Hz, 2H), 7.49 (s, 2H), 7.68 (d, *J* = 7.9 Hz, 4H), 8.92 (s, 2H), 10.17 (s, 2H) ppm; ^13^C NMR (100 MHz, DMSO-*d_6_*): δ = 20.1, 61.3, 98.7, 120.1, 124.5, 127.2, 128.1, 129.2, 129.3, 130.4, 134.5, 136.4, 144.2, 148.0, 151.3, 160.4 ppm; Elemental analysis: Found: C, 54.41; H, 3.63; N, 21.06; S, 4.07%; C_36_H_28_Cl_2_N_12_O_4_S; requires: C, 54.34; H, 3.55; N, 21.13; S, 4.03%.

***N,N'*-(sulfonylbis(1,4-phenylene))bis(7-(3,4-dichlorophenyl)-5-methyl-4,7-dihydrotetrazolo[1,5-a]pyrimidine-6-carboxamide) (Scheme 1, Product a_9_):** ^1^H NMR (400 MHz, DMSO-*d_6_*): δ = 2.39 (s, 6H, CH_3_), 6.66 (s, 2H), 7.33 (d, *J* = 7.8 Hz, 2H), 7.37 (d, *J* = 8.3 Hz, 4H), 7.45 (d, *J* = 7.8 Hz, 2H), 7.51 (s, 2H), 7.67 (d, *J* = 8.3 Hz, 4H), 8.88 (s, 2H), 10.14 (s, 2H) ppm; ^13^C NMR (100 MHz, DMSO-*d_6_*): δ = 20.3, 61.6, 98.6, 120.2, 124.6, 128.7, 129.3, 130.1, 130.5, 134.7, 136.6, 144.3, 144.8, 148.5, 151.4, 160.6 ppm; Elemental analysis: Found: C, 50.08; H, 3.09; N, 19.40; S, 3.66%; C_36_H_26_Cl_4_N_12_O_4_S; requires: C, 50.01; H, 3.03; N, 19.44; S, 3.71%.

***N,N'*-(sulfonylbis(1,4-phenylene))bis(7-(2,4-dichlorophenyl)-5-methyl-4,7-dihydrotetrazolo[1,5-a]pyrimidine-6-carboxamide) (Scheme 1, Product a_10_):** ^1^H NMR (400 MHz, DMSO-*d_6_*): δ = 2.39 (s, 6H, CH_3_), 6.69 (s, 2H), 7.32 (d, *J* = 7.9 Hz, 2H), 7.37 (d, *J* = 8.1 Hz, 4H), 7.46 (d, *J* = 7.9 Hz, 2H), 7.53 (s, 2H), 7.68 (d, *J* = 8.1 Hz, 4H), 8.76 (s, 2H), 10.23 (s, 2H) ppm; ^13^C NMR (100 MHz, DMSO-*d_6_*): δ = 20.3, 61.9, 100.2, 120.6, 124.7, 128.4, 129.1, 129.5, 130.1, 134.7, 136.7, 144.1, 144.6, 148.2, 151.3, 160.4 ppm; Elemental analysis: Found: C, 49.98; H, 3.07; N, 19.43; S, 3.64%; C_36_H_26_Cl_4_N_12_O_4_S; requires: C, 50.01; H, 3.03; N, 19.44; S, 3.71%.

***N,N'*-(sulfonylbis(1,4-phenylene))bis(7-(3,5-dichlorophenyl)-5-methyl-4,7-dihydrotetrazolo[1,5-a]pyrimidine-6-carboxamide) (Scheme 1, Product a_11_):** ^1^H NMR (400 MHz, DMSO-*d_6_*): δ = 2.39 (s, 6H, CH_3_), 6.68 (s, 2H), 7.37 (d, *J* = 8.2 Hz, 4H), 7.47 (s, 4H), 7.50 (s, 2H), 7.68 (d, *J* = 8.2 Hz, 4H), 8.85 (s, 2H), 10.19 (s, 2H) ppm; ^13^C NMR (100 MHz, DMSO-*d_6_*): δ = 20.0, 62.3, 99.2, 120.4, 124.5, 129.3, 130.4, 130.7, 134.7, 136.4, 144.3, 148.2, 151.1, 160.2 ppm; Elemental analysis: Found: C, 50.06; H, 3.13; N, 19.51; S, 3.78%; C_36_H_26_Cl_4_N_12_O_4_S; requires: C, 50.01; H, 3.03; N, 19.44; S, 3.71%.

***N,N'*-(sulfonylbis(1,4-phenylene))bis(7-(2-chlorophenyl)-5-methyl-4,7-dihydrotetrazolo[1,5-a]pyrimidine-6-carboxamide) (Scheme 1, Product a_12_):** ^1^H NMR (400 MHz, DMSO-*d_6_*): δ = 2.37 (s, 6H, CH_3_), 6.61 (s, 2H), 7.25-7.28 (m, 4H), 7.32 (d, *J* = 7.8 Hz, 2H), 7.38 (d, *J* = 8.0 Hz, 4H), 7.43 (d, *J* = 7.8 Hz, 2H), 7.68 (d, *J* = 8.0 Hz, 4H), 8.90 (s, 2H), 10.19 (s, 2H) ppm; ^13^C NMR (100 MHz, DMSO-*d_6_*): δ = 20.1, 61.7, 98.2, 120.1, 124.4, 127.1, 127.6, 128.1, 128.4, 128.9, 134.4, 136.5, 145.2, 148.4, 151.3, 160.6 ppm; Elemental analysis: Found: C, 54.38; H, 3.67; N, 21.17; S, 4.11%; C_36_H_28_Cl_2_N_12_O_4_S; requires: C, 54.34; H, 3.55; N, 21.13; S, 4.03%.

***N,N'*-(sulfonylbis(1,4-phenylene))bis(7-(furan-2-yl)-5-methyl-4,7-dihydrotetrazolo[1,5-a]pyrimidine-6-carboxamide) (Scheme 1, Product a_13_):** ^1^H NMR (400 MHz, DMSO-*d_6_*): δ = 2.34 (s, 6H, CH_3_), 5.81 (s, 2H), 6.49 (d, *J* = 6.8 Hz, 2H), 6.56 (t, *J* = 6.9 Hz, 2H), 7.38-7.41 (m, 6H), 7.68 (d, *J* = 8.2 Hz, 4H), 8.55 (s, 2H), 10.09 (s, 2H) ppm; ^13^C NMR (100 MHz, DMSO-*d_6_*): δ = 19.7, 56.7, 97.2, 104.6, 111.3, 119.7, 123.4, 124.5, 127.9, 131.4, 134.1, 148.1, 151.2, 159.7 ppm; Elemental analysis: Found: C, 54.36; H, 3.65; N, 23.71; S, 4.45%; C_32_H_26_N_12_O_6_S; requires: C, 54.39; H, 3.71; N, 23.78; S, 4.54%.

***N,N'*-(sulfonylbis(1,4-phenylene))bis(5-methyl-7-(2-oxo-2*H*-chromen-4-yl)-4,7-dihydrotetrazolo[1,5-a]pyrimidine-6-carboxamide) (Scheme 1, Product a_14_):** ^1^H NMR (400 MHz, DMSO-*d_6_*): δ = 2.39 (s, 6H, CH_3_), 6.45 (s, 2H), 6.81 (s, 2H), 6.98 (d, *J* = 8.2 Hz, 2H), 7.38 (d, *J* = 8.1 Hz, 4H), 7.43 (t, *J* = 8.2 Hz, 2H), 7.69 (d, *J* = 8.2 Hz, 4H), 7.76 (t, *J* = 8.2 Hz, 2H), 7.92 (d, *J* = 8.1 Hz, 2H), 8.91 (s, 2H), 10.29 (s, 2H) ppm; ^13^C NMR (100 MHz, DMSO-*d_6_*): δ = 20.7, 63.7, 98.1, 102.3, 106.6, 119.7, 124.5, 127.8, 128.6, 129.3, 131.4, 133.7, 134.7, 138.9, 148.2, 151.2, 155.6, 161.7, 173.8 ppm; Elemental analysis: Found: C, 58.43; H, 3.55; N, 19.49; S, 3.68%; C_42_H_30_N_12_O_8_S; requires: C, 58.47; H, 3.50; N, 19.48; S, 3.72%.

**7-(9-ethyl-9*H*-carbazol-2-yl)-*N*-(4-((4-(7-(9-ethyl-9*H*-carbazol-3-yl)-5-methyl-4,7-dihydrotetrazolo[1,5-a]pyrimidine-6-carboxamido)phenyl)sulfonyl)phenyl)-5-methyl-4,7-dihydrotetrazolo[1,5-a]pyrimidine-6-carboxamide (Scheme 1, Product a_15_):** ^1^H NMR (400 MHz, DMSO-*d_6_*): δ = 0.97 (t, *J* = 6.4 Hz, 6H), 2.37 (s, 6H, CH_3_), 3.49 (q, *J* = 6.4 Hz, 4H), 6.21 (s, 2H), 6.84 (d, *J* = 8.0 Hz, 2H), 7.02 (s, 2H), 7.18 (t, *J* = 8.0 Hz, 2H), 7.24-7.27 (m, 4H), 7.36-7.40 (m, 6H), 7.67 (d, *J* = 8.1 Hz, 4H), 7.82 (d, *J* = 8.1 Hz, 2H), 8.89 (s, 2H), 10.11 (s, 2H) ppm; ^13^C NMR (100 MHz, DMSO-*d_6_*): δ = 15.3, 20.8, 34.9, 64.7, 98.6, 107.1, 108.6, 111.8, 112.3, 114.9, 115.6, 123.5, 126.7, 127.4, 127.8, 128.6, 129.3, 134.4, 136.4, 137.1, 137.8, 148.2, 151.4, 162.9 ppm; Elemental analysis: Found: C, 65.07; H, 4.69; N, 20.45; S, 3.38%; C_52_H_44_N_14_O_4_S; requires: C, 64.99; H, 4.61; N, 20.40; S, 3.34%.

**5-methyl-*N*-(4-((4-(5-methyl-7-(9-methyl-9*H*-carbazol-2-yl)-4,7-dihydrotetrazolo[1,5-a]pyrimidine-6-carboxamido)phenyl)sulfonyl)phenyl)-7-(9-methyl-9*H*-carbazol-3-yl)-4,7-dihydrotetrazolo[1,5-a]pyrimidine-6-carboxamide (Scheme 1, Product a_16_):** ^1^H NMR (400 MHz, DMSO-*d_6_*): δ = 2.37 (s, 6H, CH_3_), 3.43 (s, 6H), 6.23 (s, 2H), 6.85 (d, *J* = 8.2 Hz, 2H), 7.04 (s, 2H), 7.18 (t, *J* = 8.2 Hz, 2H), 7.23-7.27 (m, 4H), 7.35-7.39 (m, 6H), 7.67 (d, *J* = 8.0 Hz, 4H), 7.81 (d, *J* = 8.2 Hz, 2H), 8.96 (s, 2H), 10.18 (s, 2H) ppm; ^13^C NMR (100 MHz, DMSO-*d_6_*): δ = 20.6, 34.1, 64.7, 97.9, 107.0, 108.3, 111.2, 112.4, 114.7, 115.1, 123.9, 126.6, 127.4, 127.7, 128.6, 129.2, 134.4, 136.3, 136.8, 137.4, 148.2, 151.1, 162.2 ppm; Elemental analysis: Found: C, 64.29; H, 4.37; N, 21.06; S, 3.38%; C_50_H_40_N_14_O_4_S; requires: C, 64.37; H, 4.32; N, 21.02; S, 3.44%.


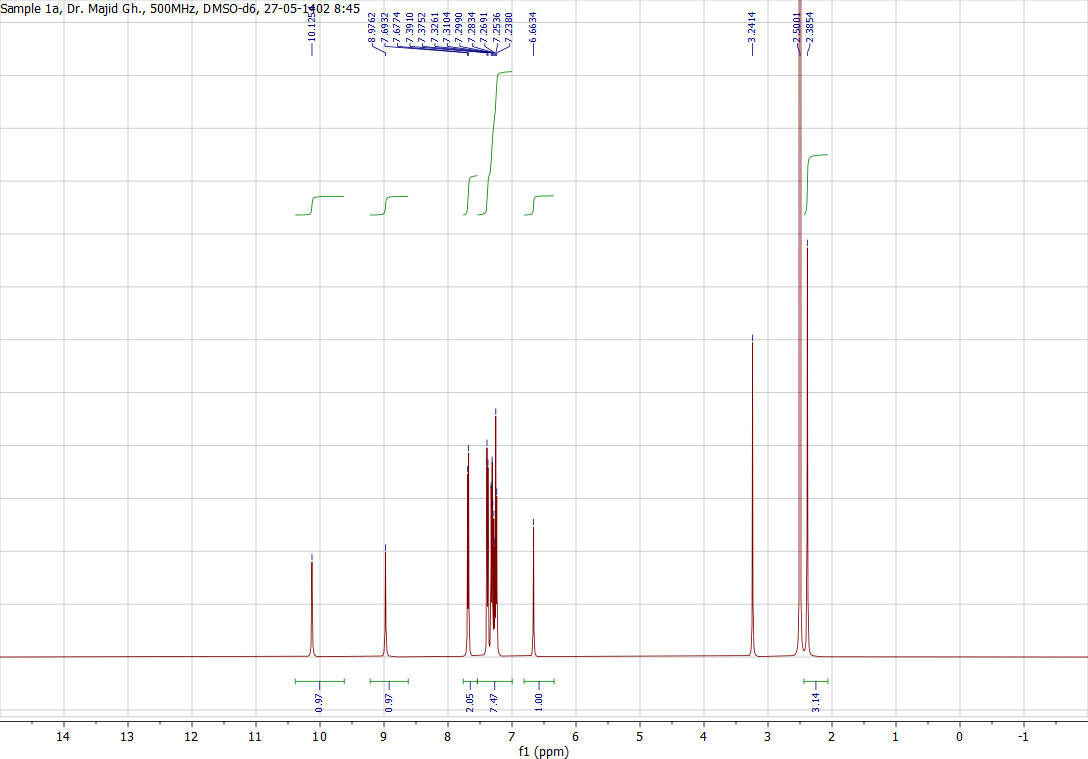


**Supplementary Figure 1:** ^1^H-NMR spectrum of *N,N'*-(sulfonylbis(1,4-phenylene))bis(5-methyl-7-phenyl-4,7-dihydrotetrazolo[1,5-a]pyrimidine-6-carboxamide) (Scheme 1, Product a_1_)


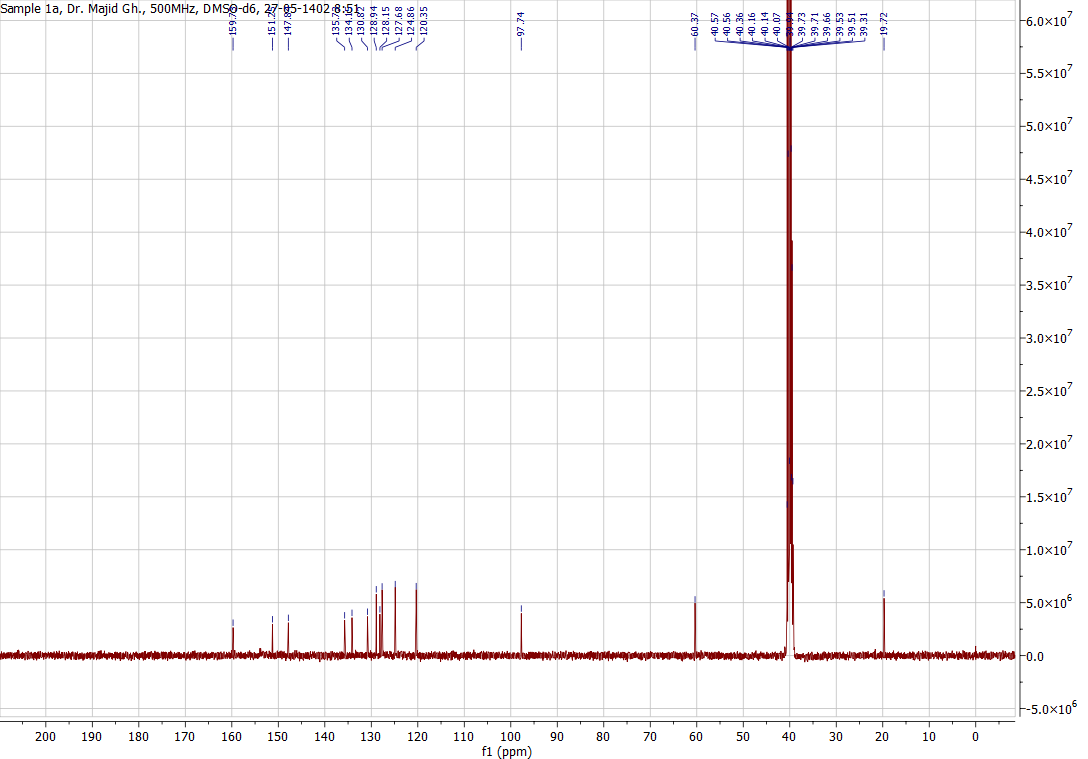


**Supplementary Figure 2:** ^13^C-NMR spectrum of *N,N'*-(sulfonylbis(1,4-phenylene))bis(5-methyl-7-phenyl-4,7-dihydrotetrazolo[1,5-a]pyrimidine-6-carboxamide) (Scheme 1, Product a_1_)


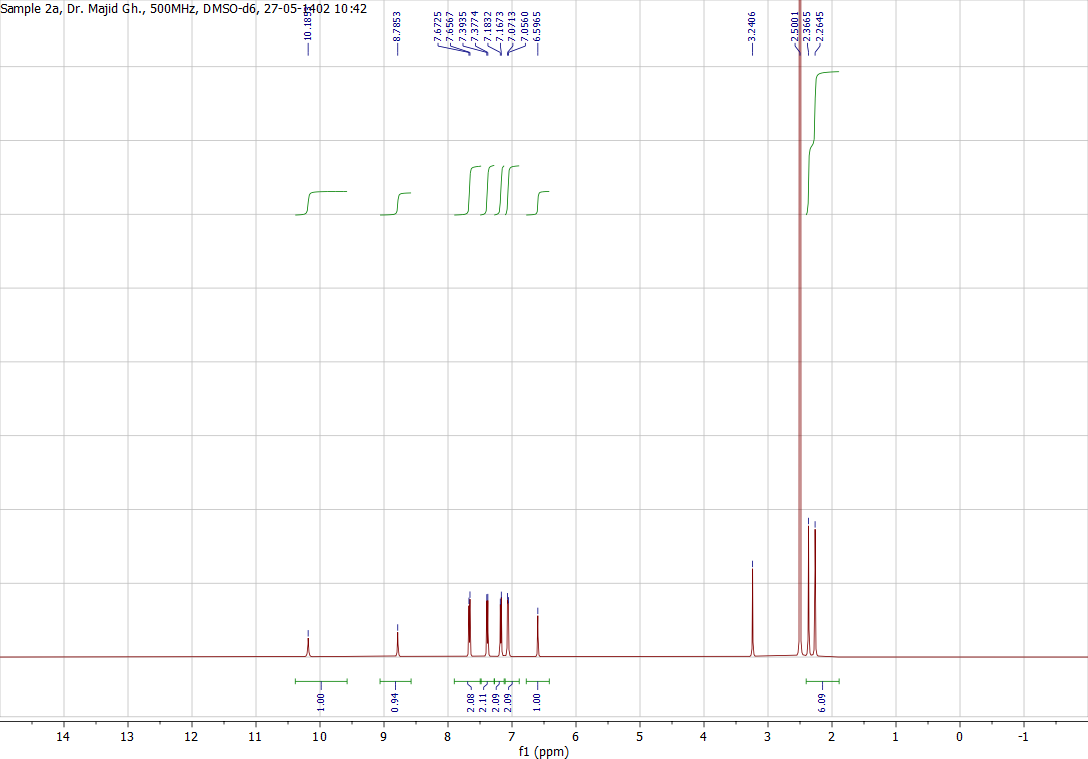


**Supplementary Figure 3:** ^1^H-NMR spectrum of *N,N'*-(sulfonylbis(1,4-phenylene))bis(5-methyl-7-(*p*-tolyl)-4,7-dihydrotetrazolo[1,5-a]pyrimidine-6-carboxamide) (Scheme 1, Product a_2_)


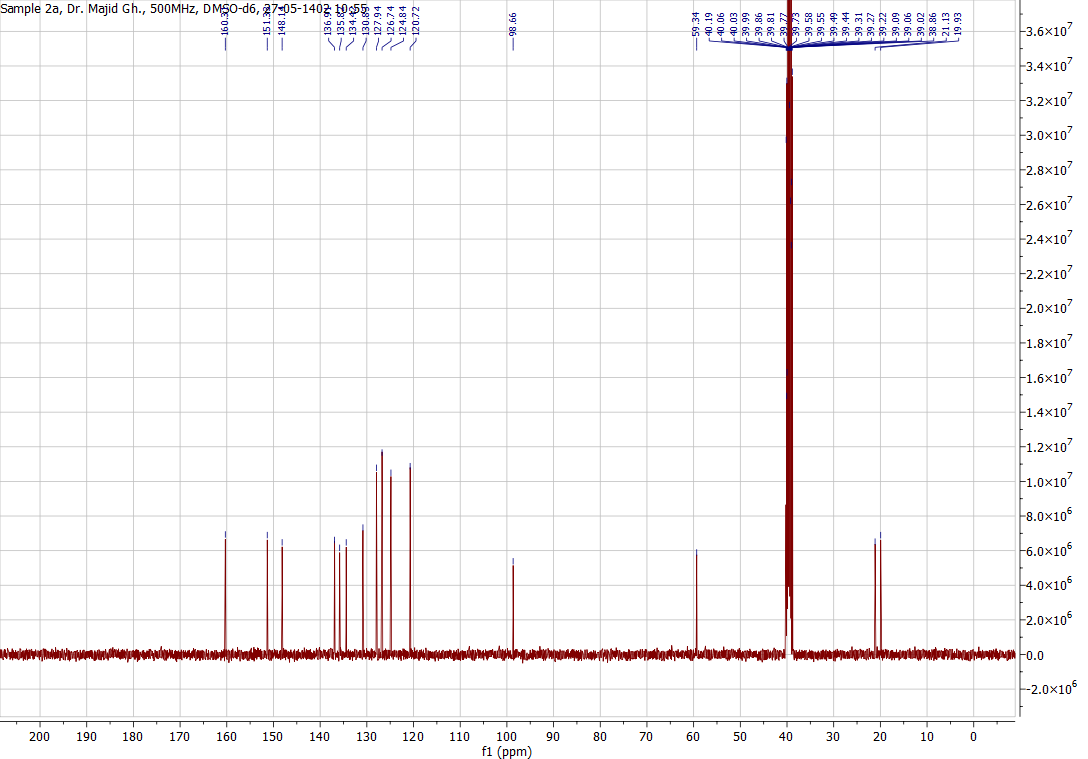


**Supplementary Figure 4:** ^13^C-NMR spectrum of *N,N'*-(sulfonylbis(1,4-phenylene))bis(5-methyl-7-(*p*-tolyl)-4,7-dihydrotetrazolo[1,5-a]pyrimidine-6-carboxamide) (Scheme 1, Product a_2_)


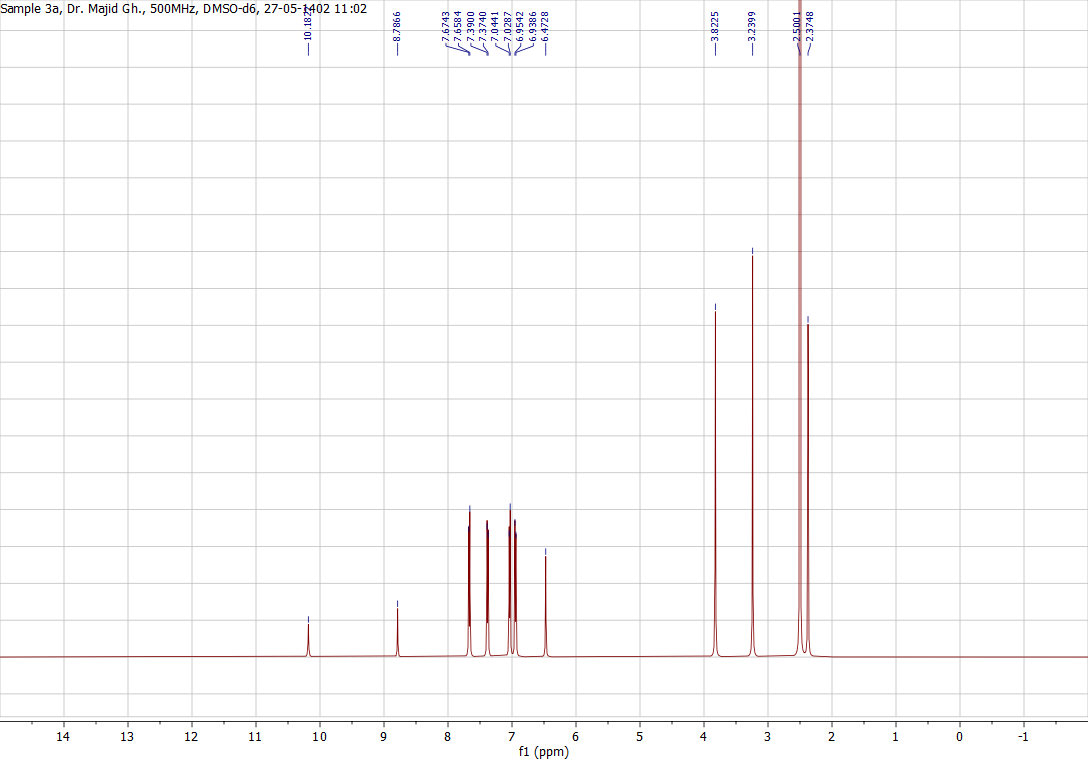


**Supplementary Figure 5:** ^1^H-NMR spectrum of *N,N'*-(sulfonylbis(1,4-phenylene))bis(7-(4-methoxyphenyl)-5-methyl-4,7-dihydrotetrazolo[1,5-a]pyrimidine-6-carboxamide) (Scheme 1, Product a_3_)


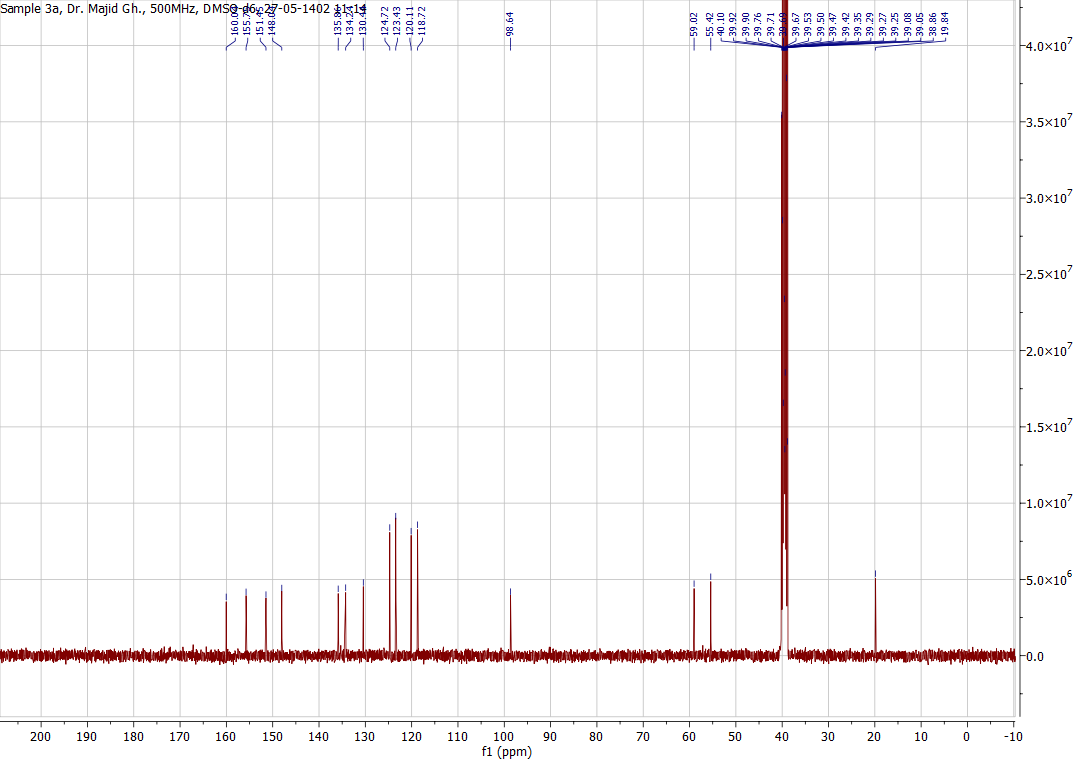


**Supplementary Figure 6:** ^13^C-NMR spectrum of *N,N'*-(sulfonylbis(1,4-phenylene))bis(7-(4-methoxyphenyl)-5-methyl-4,7-dihydrotetrazolo[1,5-a]pyrimidine-6-carboxamide) (Scheme 1, Product a_3_)


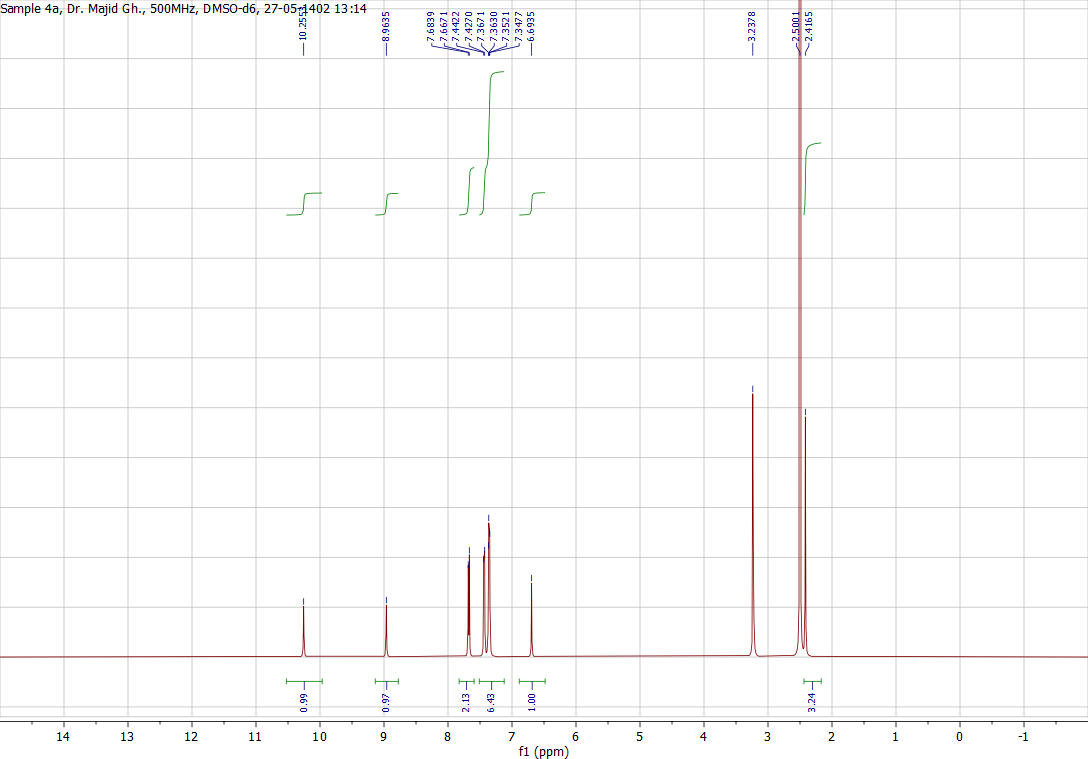


**Supplementary Figure 7:** ^1^H-NMR spectrum of *N,N'*-(sulfonylbis(1,4-phenylene))bis(7-(4-chlorophenyl)-5-methyl-4,7-dihydrotetrazolo[1,5-a]pyrimidine-6-carboxamide) (Scheme 1, Product a_4_)


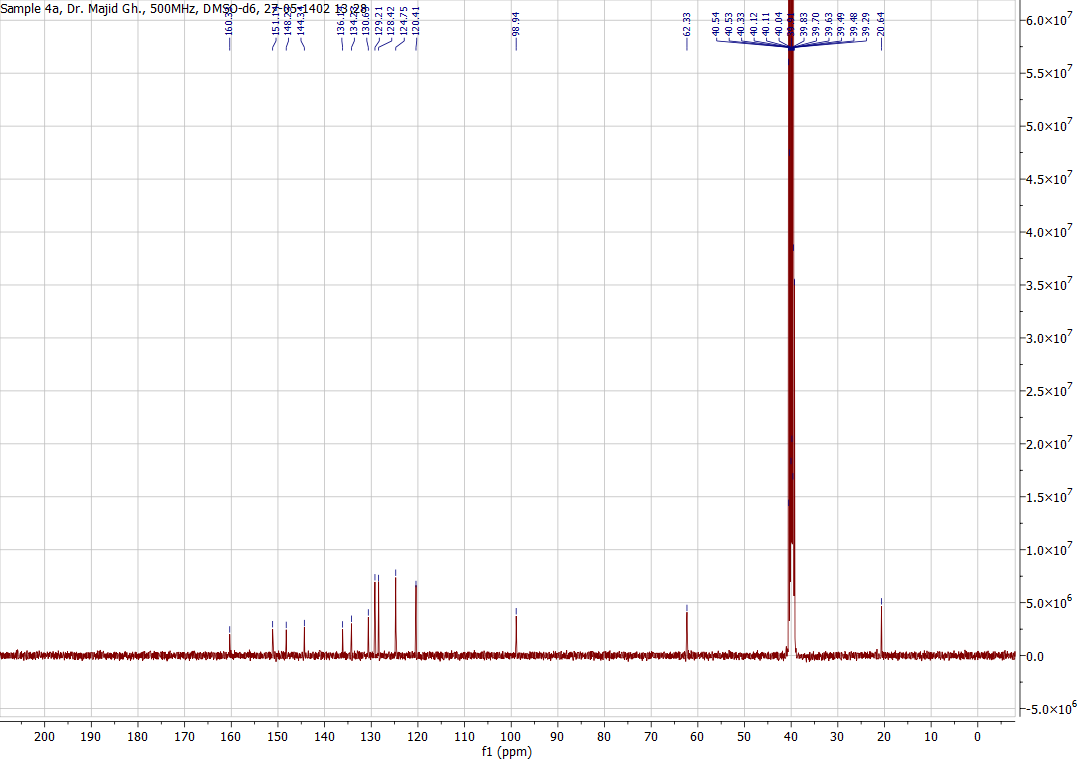


**Supplementary Figure 8:** ^13^C-NMR spectrum of *N,N'*-(sulfonylbis(1,4-phenylene))bis(7-(4-chlorophenyl)-5-methyl-4,7-dihydrotetrazolo[1,5-a]pyrimidine-6-carboxamide) (Scheme 1, Product a_4_)


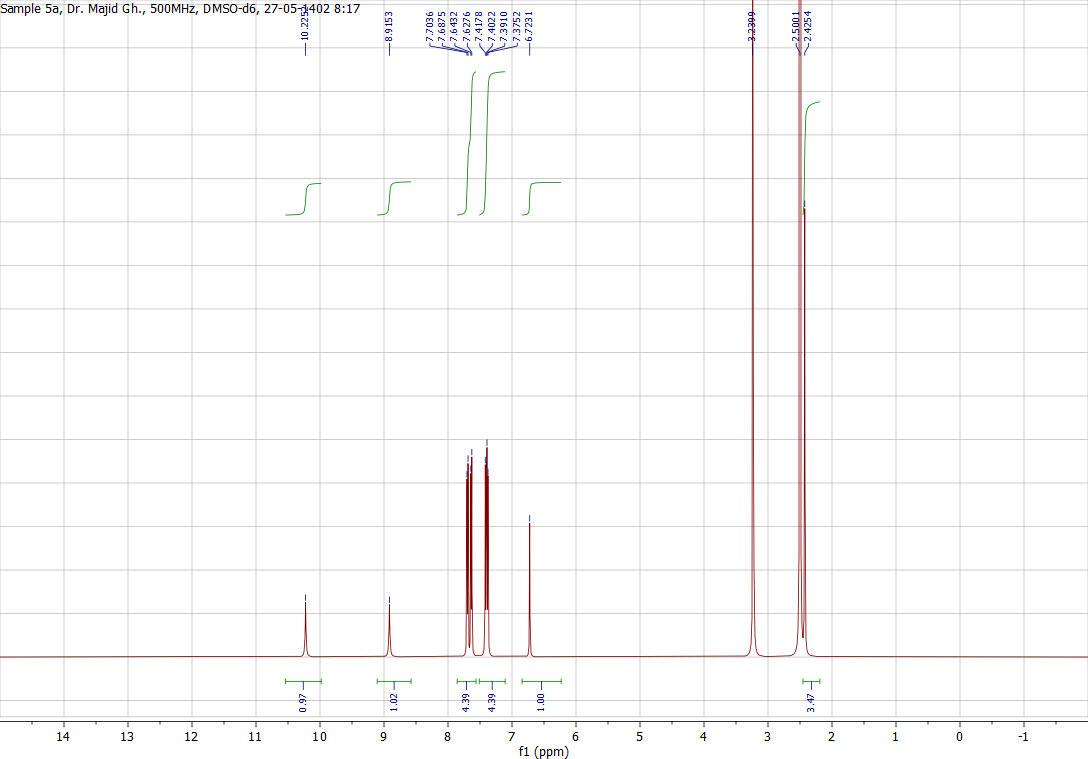


**Supplementary Figure 9:** ^1^H-NMR spectrum of *N,N'*-(sulfonylbis(1,4-phenylene))bis(7-(4-bromophenyl)-5-methyl-4,7-dihydrotetrazolo[1,5-a]pyrimidine-6-carboxamide) (Scheme 1, Product a_5_)


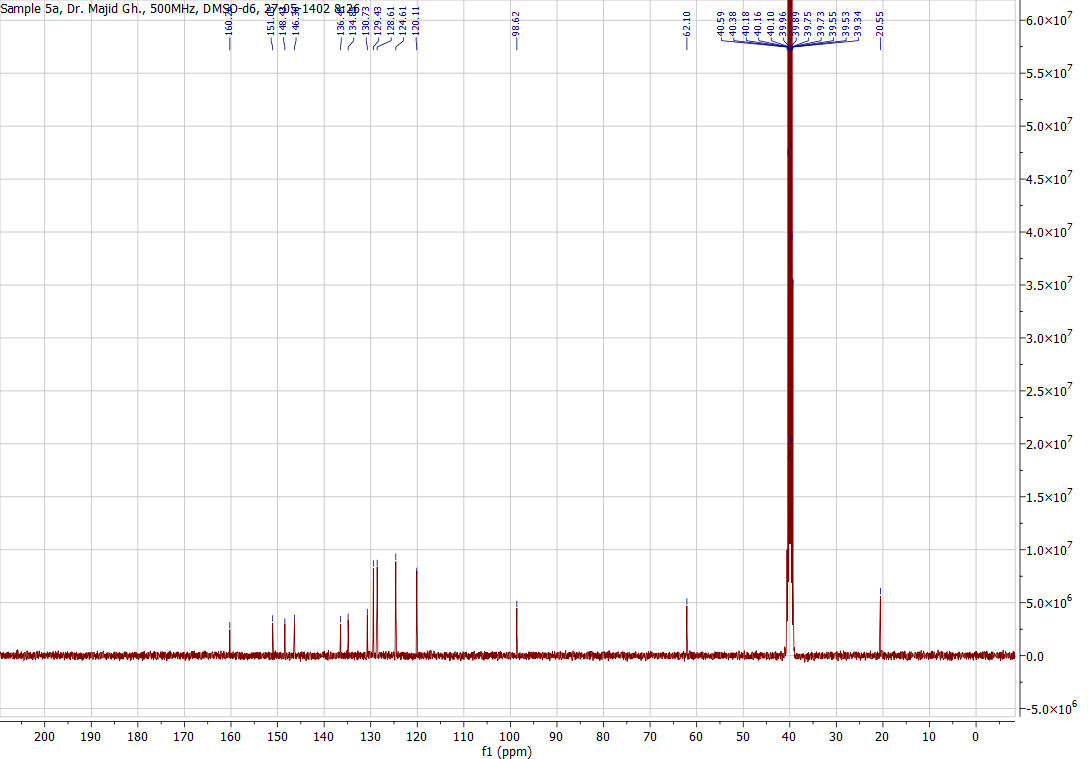


**Supplementary Figure 10:** ^13^C-NMR spectrum of *N,N'*-(sulfonylbis(1,4-phenylene))bis(7-(4-bromophenyl)-5-methyl-4,7-dihydrotetrazolo[1,5-a]pyrimidine-6-carboxamide) (Scheme 1, Product a_5_)


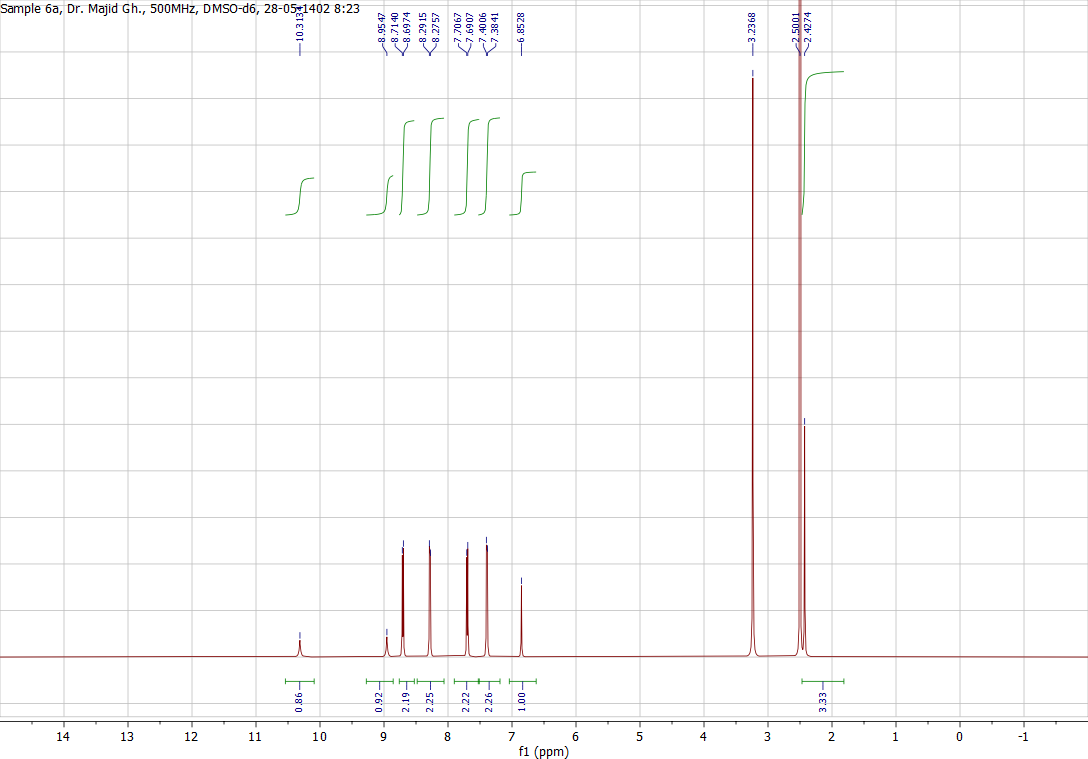


**Supplementary Figure 11:** ^1^H-NMR spectrum of *N,N'*-(sulfonylbis(1,4-phenylene))bis(5-methyl-7-(4-nitrophenyl)-4,7-dihydrotetrazolo[1,5-a]pyrimidine-6-carboxamide) (Scheme 1, Product a_6_)


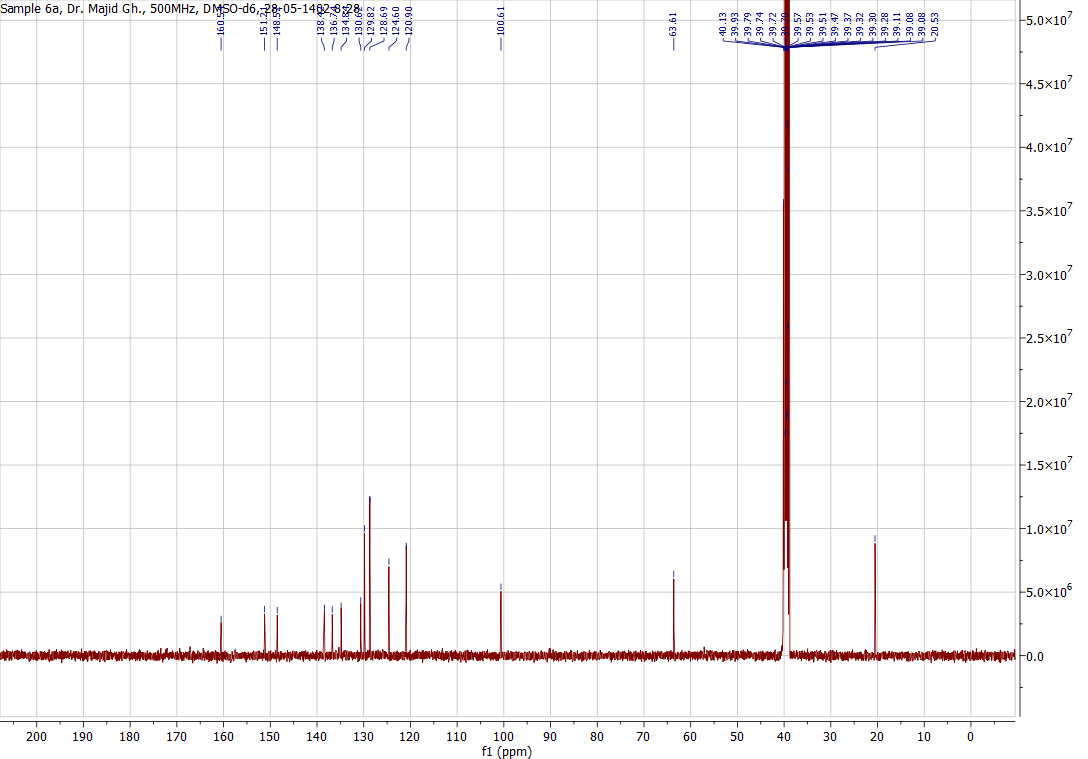


**Supplementary Figure 12:** ^13^C-NMR spectrum of *N,N'*-(sulfonylbis(1,4-phenylene))bis(5-methyl-7-(4-nitrophenyl)-4,7-dihydrotetrazolo[1,5-a]pyrimidine-6-carboxamide) (Scheme 1, Product a_6_)


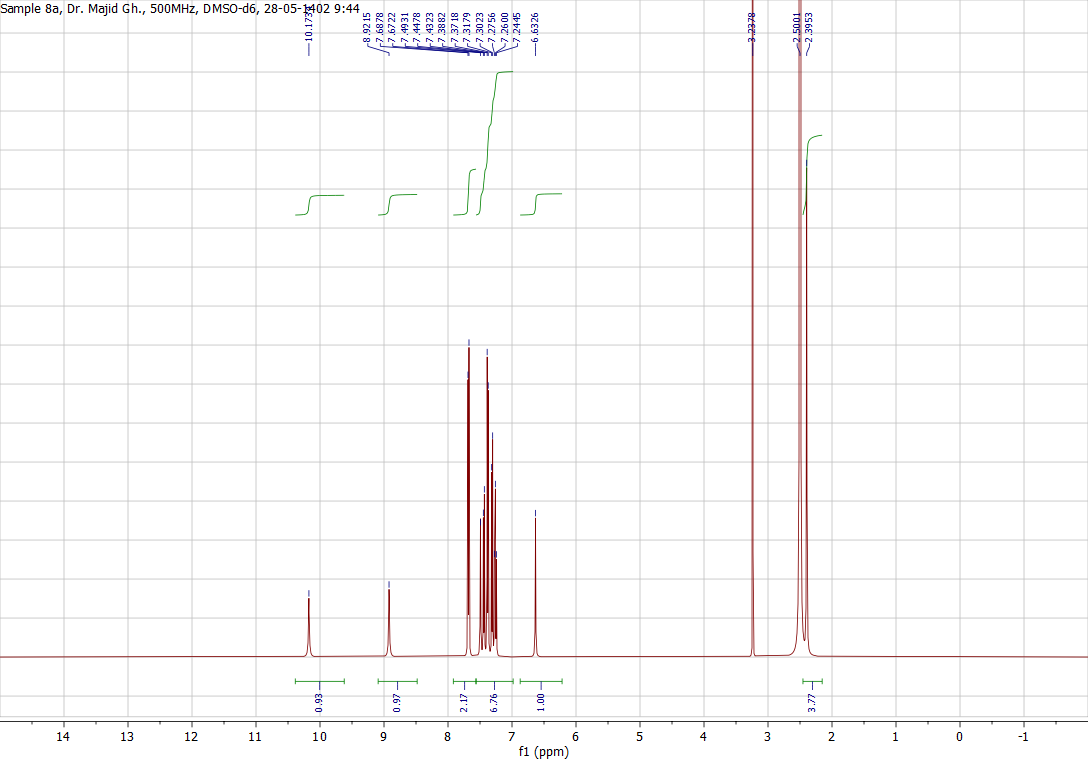


**Supplementary Figure 13:** ^1^H-NMR spectrum of *N,N'*-(sulfonylbis(1,4-phenylene))bis(5-methyl-7-(3-nitrophenyl)-4,7-dihydrotetrazolo[1,5-a]pyrimidine-6-carboxamide) (Scheme 1, Product a_7_)


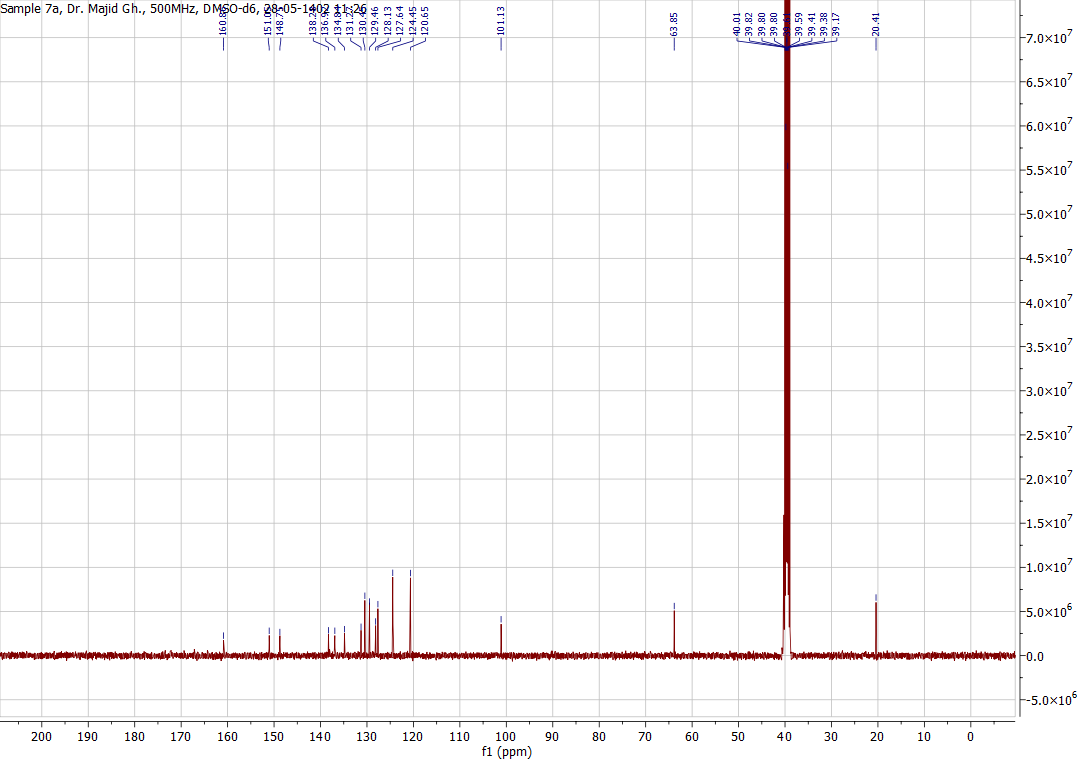


**Supplementary Figure 14:** ^13^C-NMR spectrum of *N,N'*-(sulfonylbis(1,4-phenylene))bis(5-methyl-7-(3-nitrophenyl)-4,7-dihydrotetrazolo[1,5-a]pyrimidine-6-carboxamide) (Scheme 1, Product a_7_)


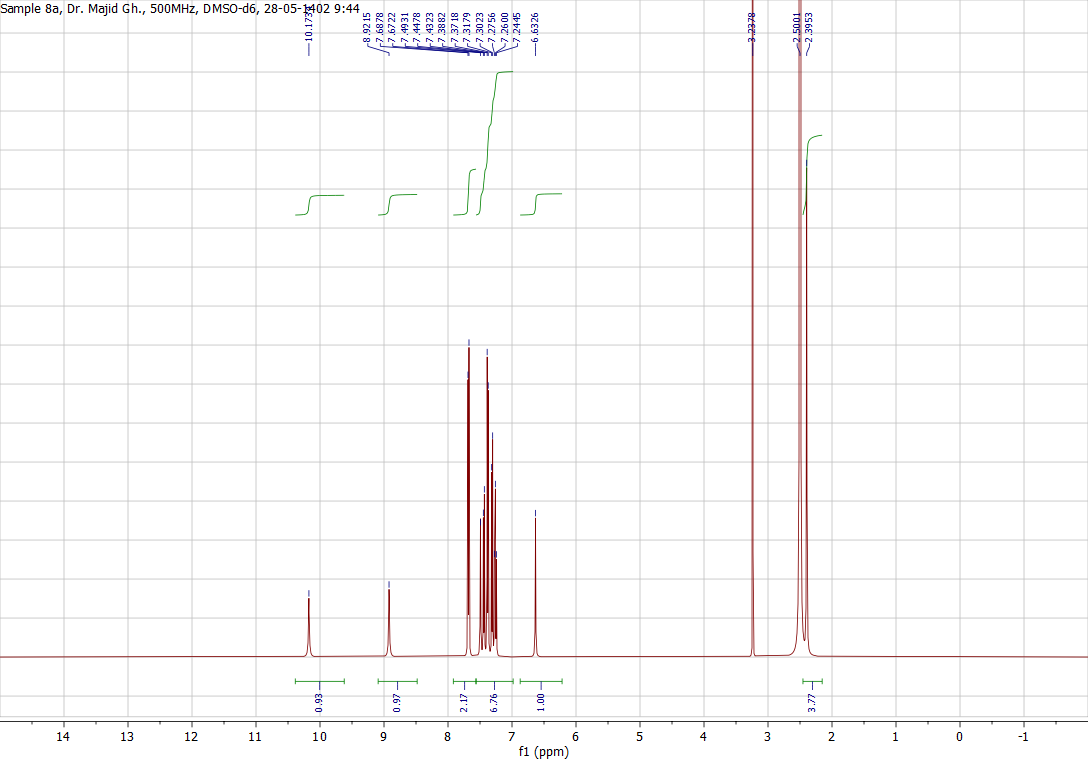


**Supplementary Figure 15:** ^1^H-NMR spectrum of *N,N'*-(sulfonylbis(1,4-phenylene))bis(7-(3-chlorophenyl)-5-methyl-4,7-dihydrotetrazolo[1,5-a]pyrimidine-6-carboxamide) (Scheme 1, Product a_8_)


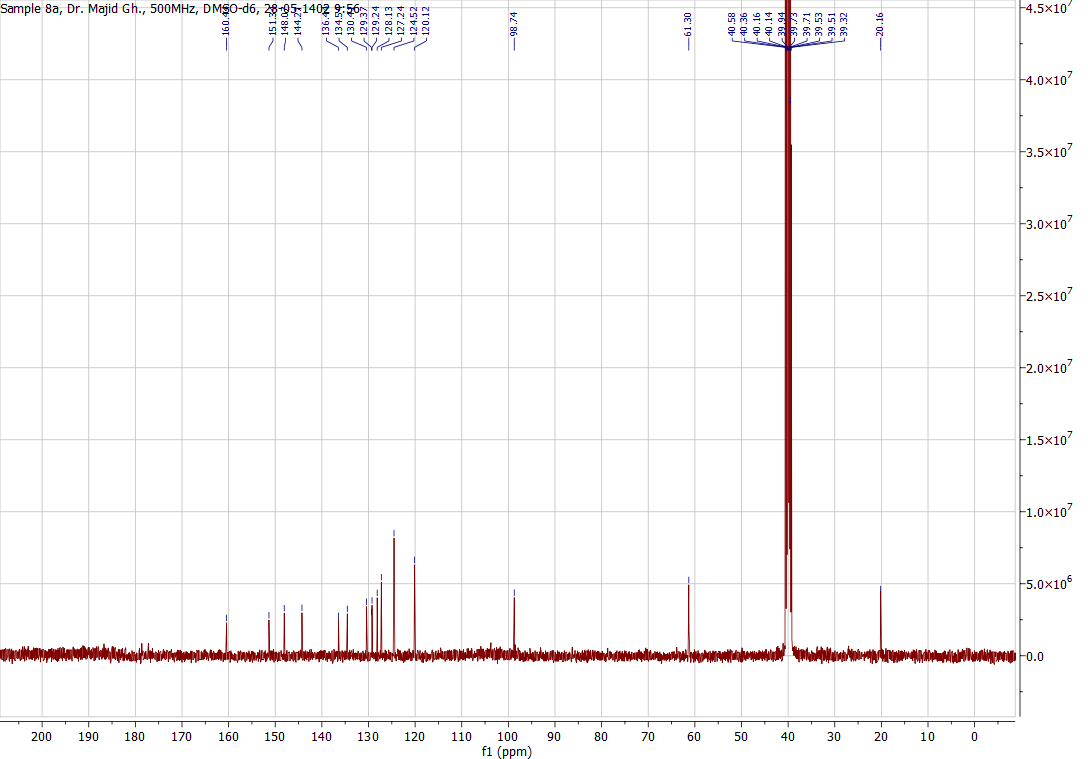


**Supplementary Figure 16:** ^13^C-NMR spectrum of *N,N'*-(sulfonylbis(1,4-phenylene))bis(7-(3-chlorophenyl)-5-methyl-4,7-dihydrotetrazolo[1,5-a]pyrimidine-6-carboxamide) (Scheme 1, Product a_8_)


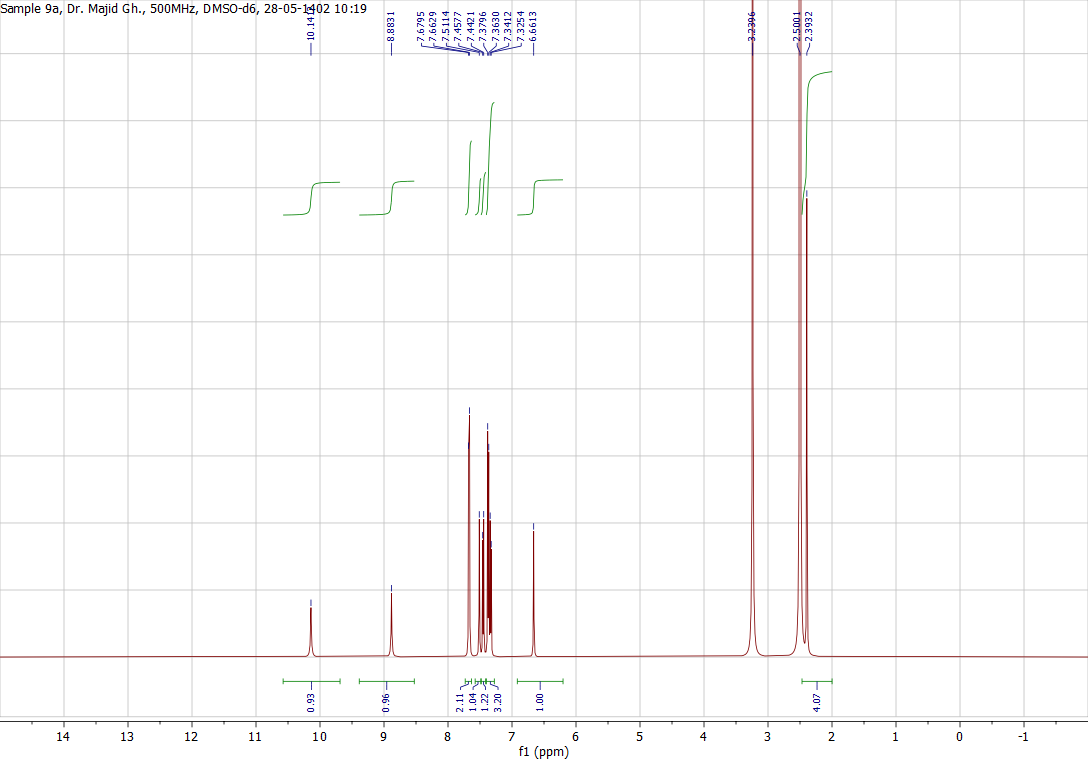


**Supplementary Figure 17:** ^1^H-NMR spectrum of *N,N'*-(sulfonylbis(1,4-phenylene))bis(7-(3,4-dichlorophenyl)-5-methyl-4,7-dihydrotetrazolo[1,5-a]pyrimidine-6-carboxamide) (Scheme 1, Product a_9_)


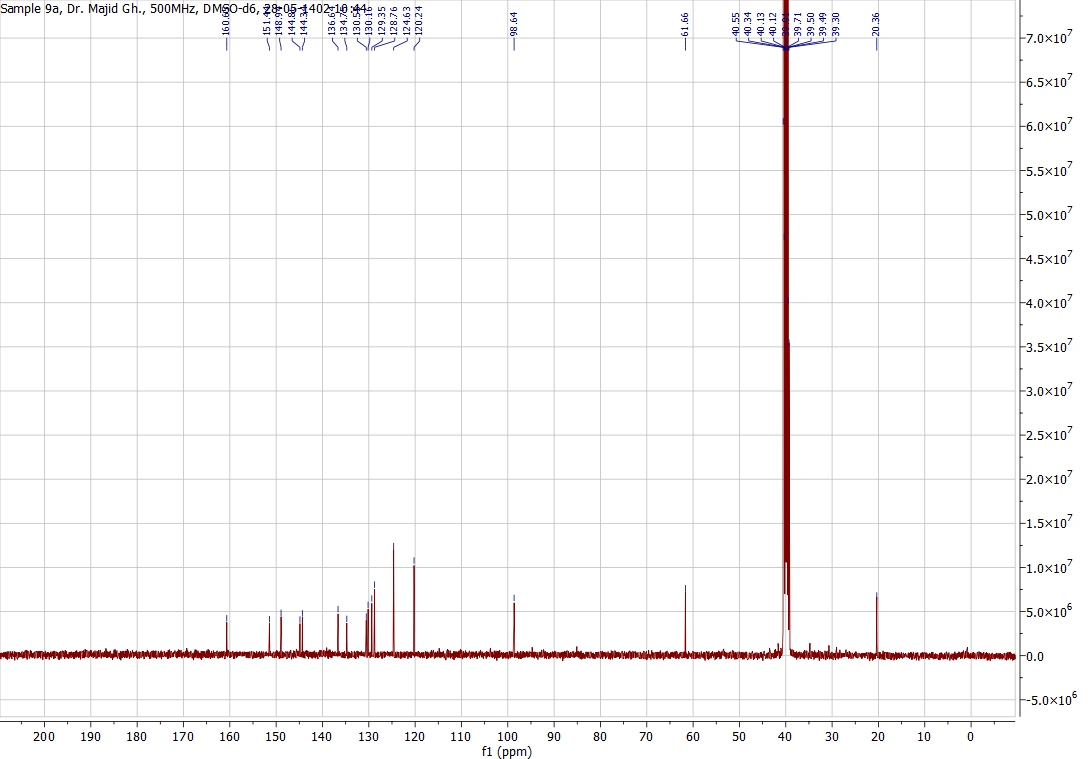


**Supplementary Figure 18:** ^13^C-NMR spectrum of *N,N'*-(sulfonylbis(1,4-phenylene))bis(7-(3,4-dichlorophenyl)-5-methyl-4,7-dihydrotetrazolo[1,5-a]pyrimidine-6-carboxamide) (Scheme 1, Product a_9_)


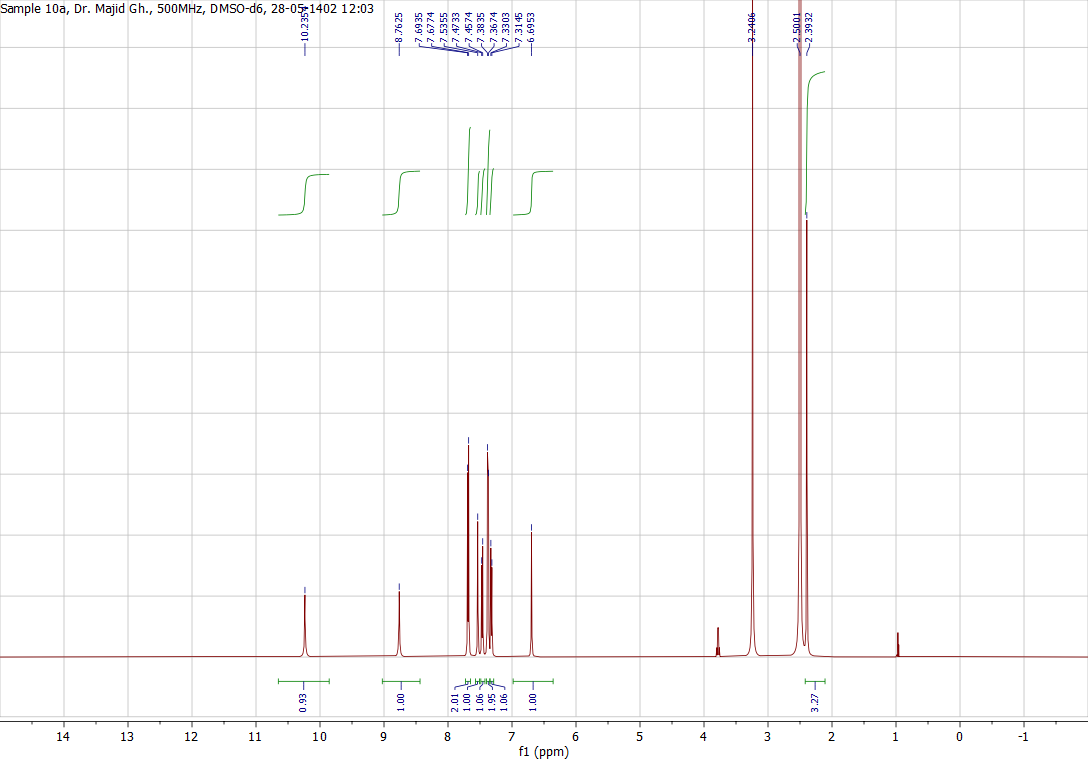


**Supplementary Figure 19:** ^1^H-NMR spectrum of *N,N'*-(sulfonylbis(1,4-phenylene))bis(7-(2,4-dichlorophenyl)-5-methyl-4,7-dihydrotetrazolo[1,5-a]pyrimidine-6-carboxamide) (Scheme 1, Product a_10_)


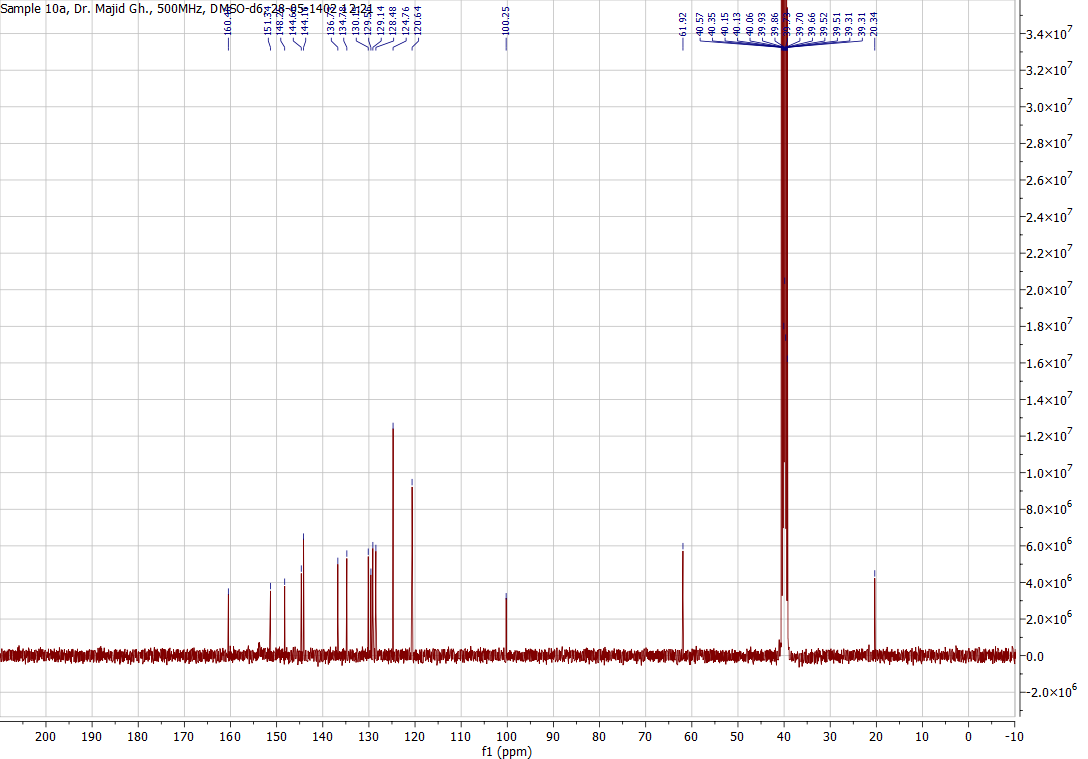


**Supplementary Figure 20:** ^13^C-NMR spectrum of *N,N'*-(sulfonylbis(1,4-phenylene))bis(7-(2,4-dichlorophenyl)-5-methyl-4,7-dihydrotetrazolo[1,5-a]pyrimidine-6-carboxamide) (Scheme 1, Product a_10_)


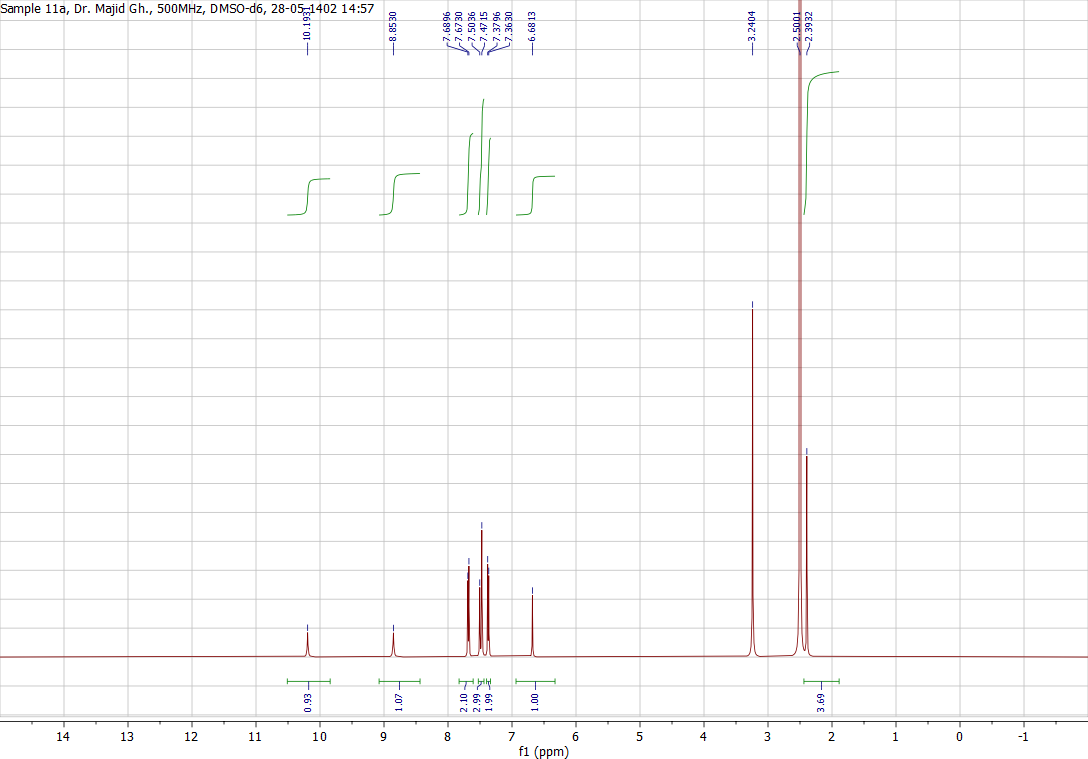


**Supplementary Figure 21:** ^1^H-NMR spectrum of *N,N'*-(sulfonylbis(1,4-phenylene))bis(7-(3,5-dichlorophenyl)-5-methyl-4,7-dihydrotetrazolo[1,5-a]pyrimidine-6-carboxamide) (Scheme 1, Product a_11_)


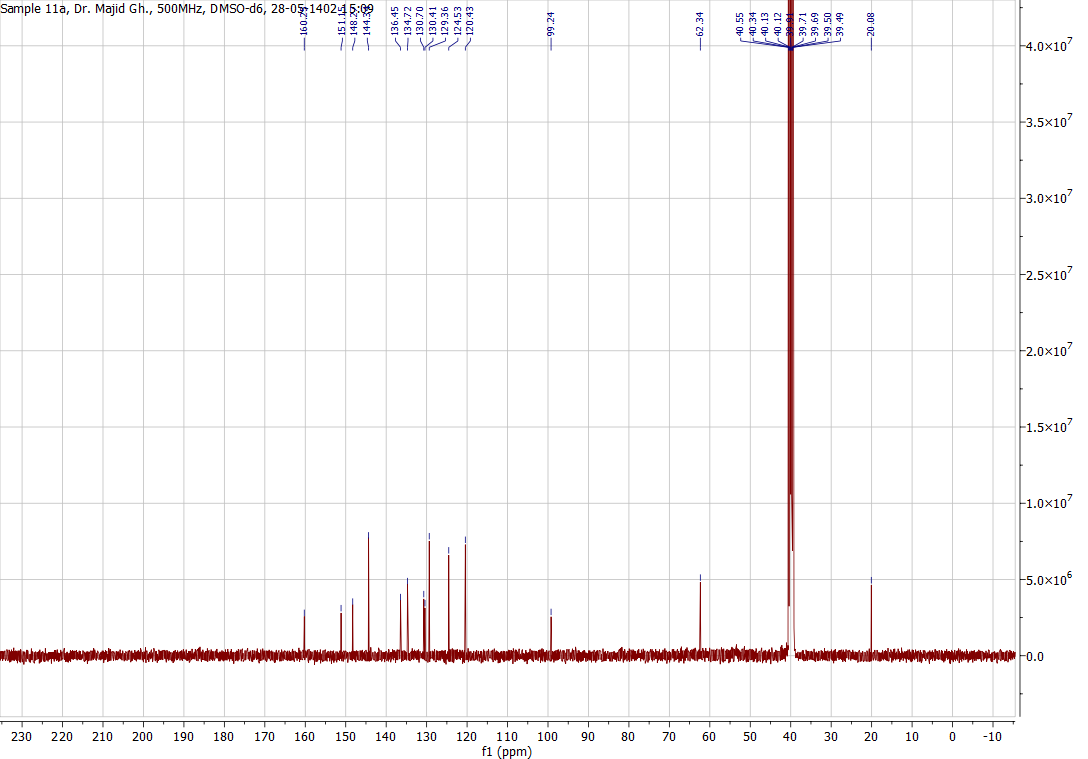


**Supplementary Figure 22:** ^13^C-NMR spectrum of *N,N'*-(sulfonylbis(1,4-phenylene))bis(7-(3,5-dichlorophenyl)-5-methyl-4,7-dihydrotetrazolo[1,5-a]pyrimidine-6-carboxamide) (Scheme 1, Product a_11_)


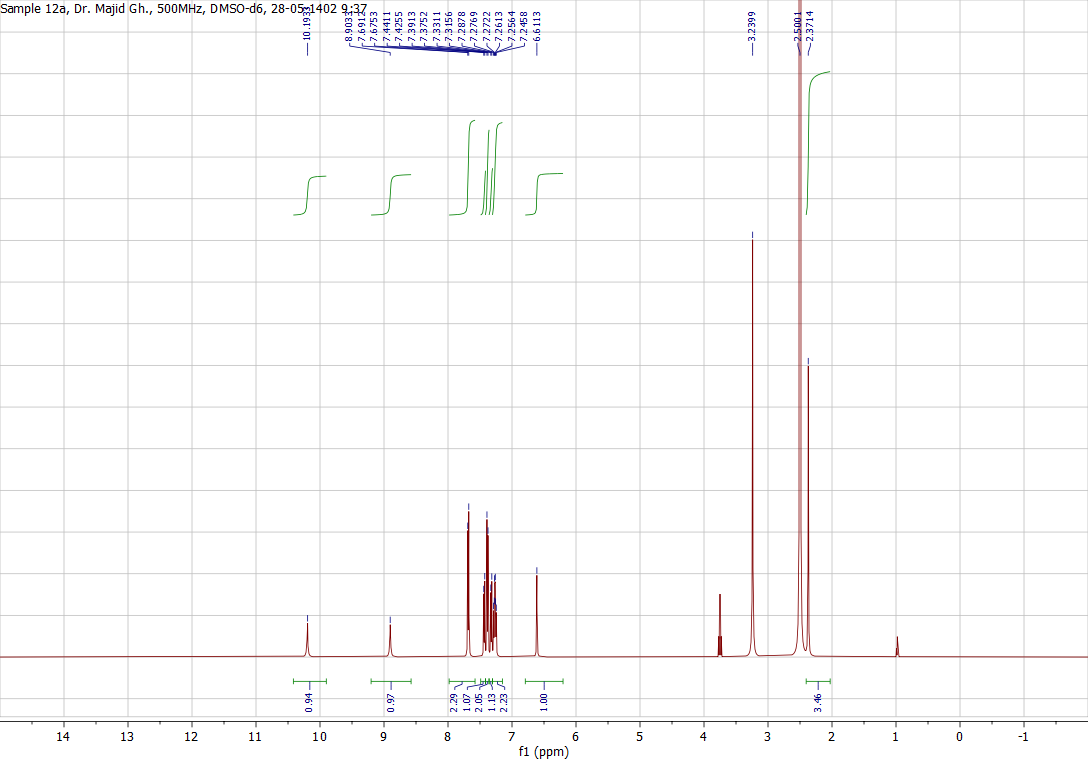


**Supplementary Figure 23:** ^1^H-NMR spectrum of *N,N'*-(sulfonylbis(1,4-phenylene))bis(7-(2-chlorophenyl)-5-methyl-4,7-dihydrotetrazolo[1,5-a]pyrimidine-6-carboxamide) (Scheme 1, Product a_12_)


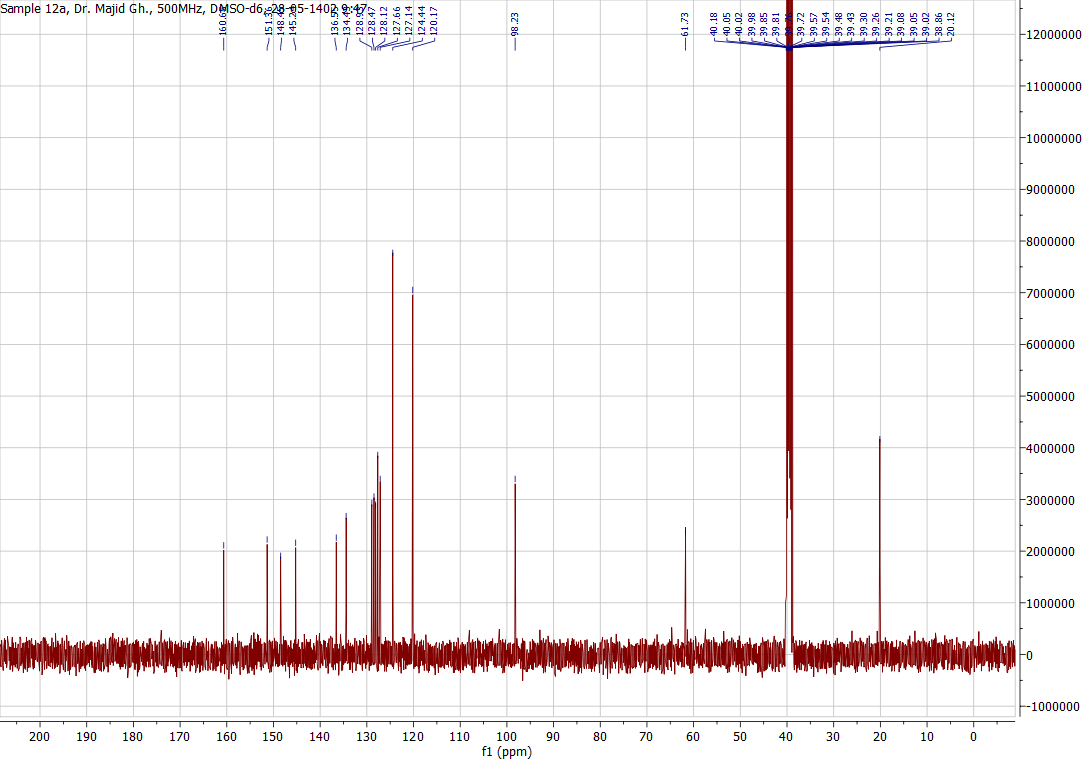


**Supplementary Figure 24:** ^13^C-NMR spectrum of *N,N'*-(sulfonylbis(1,4-phenylene))bis(7-(2-chlorophenyl)-5-methyl-4,7-dihydrotetrazolo[1,5-a]pyrimidine-6-carboxamide) (Scheme 1, Product a_12_)


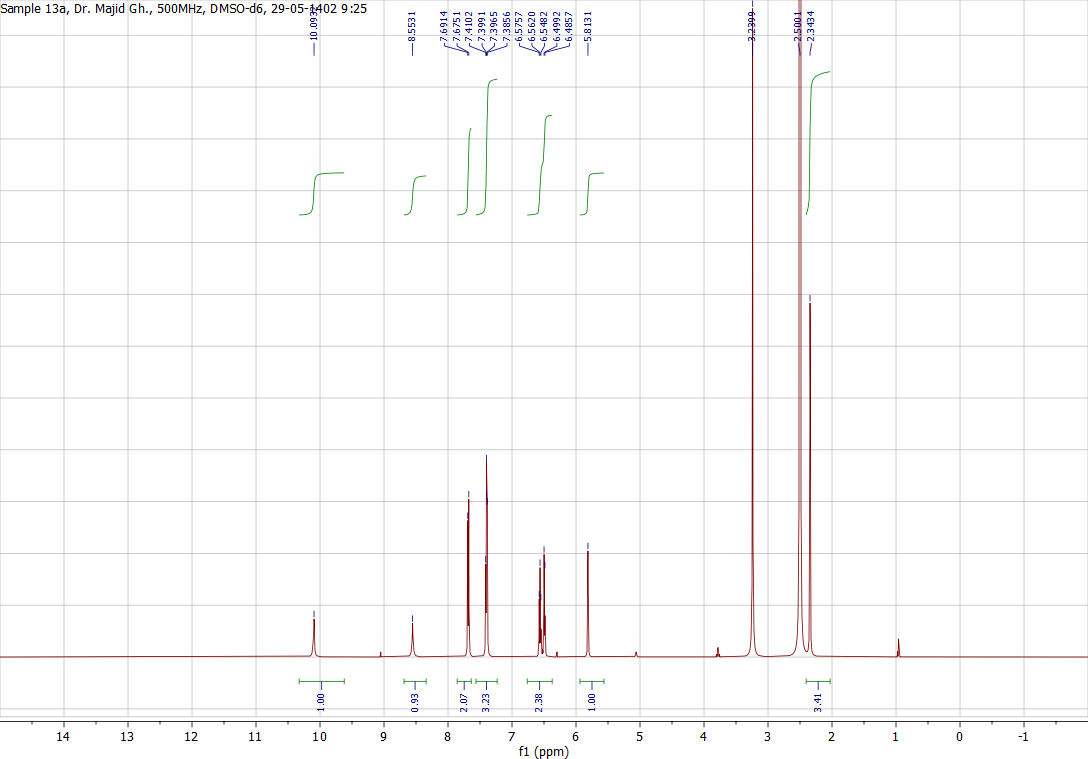


**Supplementary Figure 25:** ^1^H-NMR spectrum of *N,N'*-(sulfonylbis(1,4-phenylene))bis(7-(furan-2-yl)-5-methyl-4,7-dihydrotetrazolo[1,5-a]pyrimidine-6-carboxamide) (Scheme 1, Product a_13_)


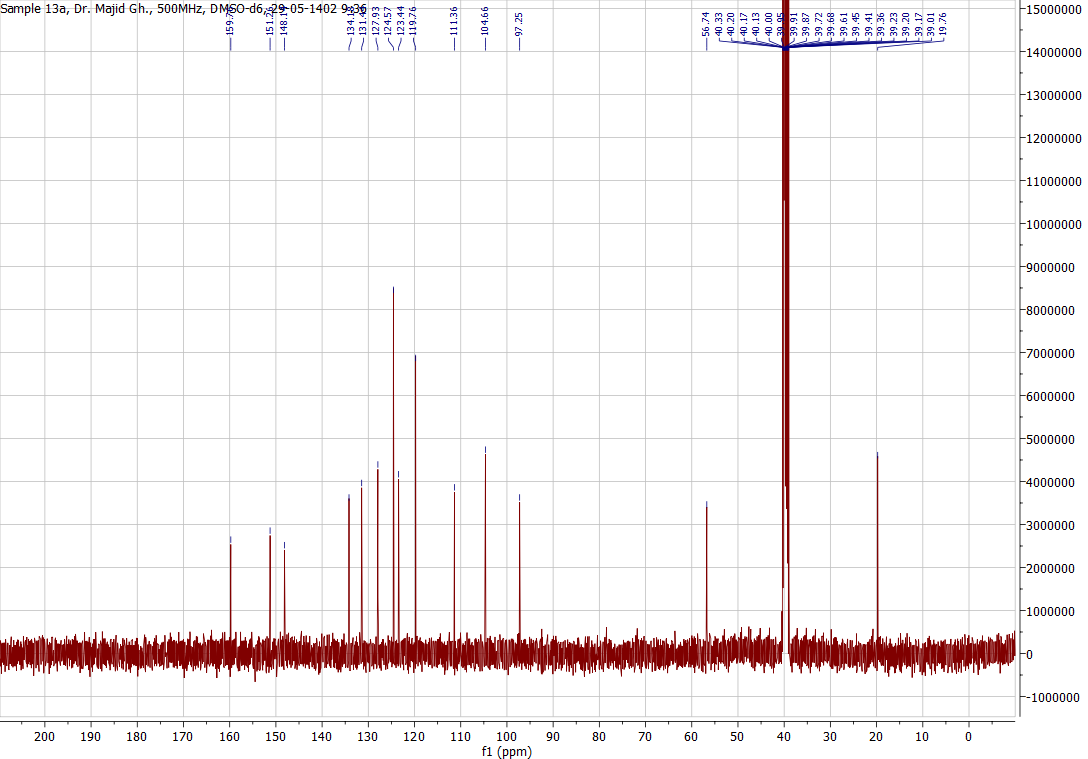


**Supplementary Figure 26:** ^13^C-NMR spectrum of *N,N'*-(sulfonylbis(1,4-phenylene))bis(7-(furan-2-yl)-5-methyl-4,7-dihydrotetrazolo[1,5-a]pyrimidine-6-carboxamide) (Scheme 1, Product a_13_)


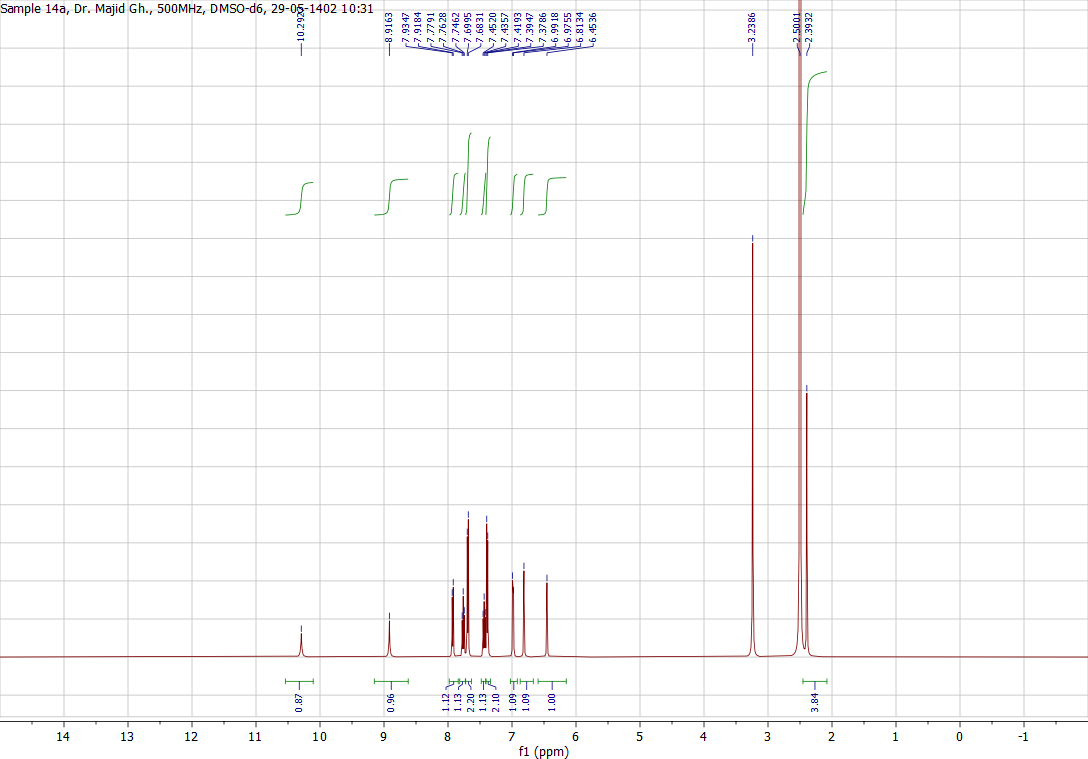


**Supplementary Figure 27:** ^1^H-NMR spectrum of *N,N'*-(sulfonylbis(1,4-phenylene))bis(5-methyl-7-(2-oxo-2*H*-chromen-4-yl)-4,7-dihydrotetrazolo[1,5-a]pyrimidine-6-carboxamide) (Scheme 1, Product a_14_)


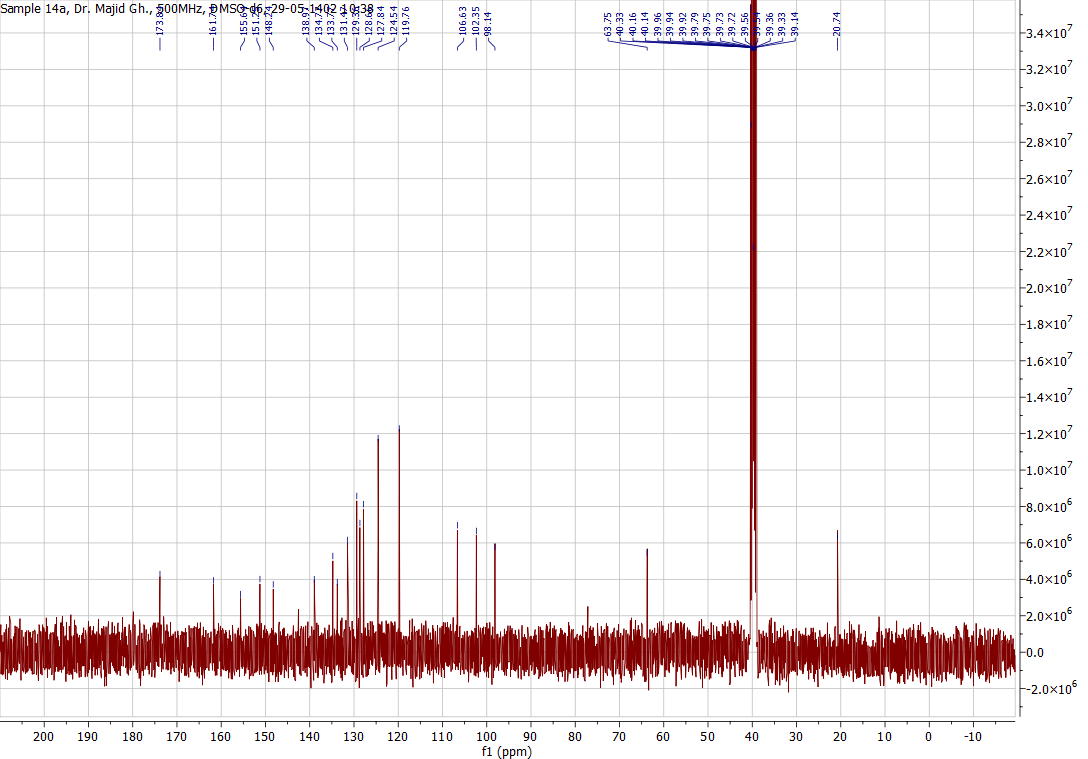


**Supplementary Figure 28:** ^13^C-NMR spectrum of *N,N'*-(sulfonylbis(1,4-phenylene))bis(5-methyl-7-(2-oxo-2*H*-chromen-4-yl)-4,7-dihydrotetrazolo[1,5-a]pyrimidine-6-carboxamide) (Scheme 1, Product a_14_)


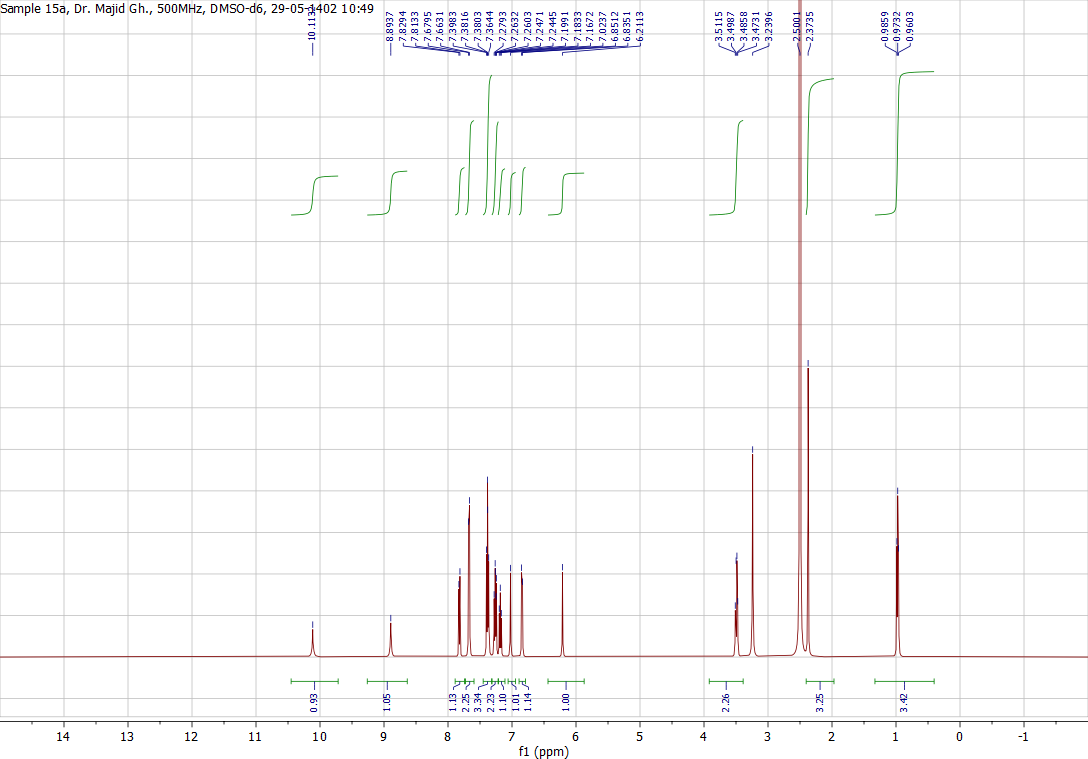


**Supplementary Figure 29:** ^1^H-NMR spectrum of 7-(9-ethyl-9*H*-carbazol-2-yl)-*N*-(4-((4-(7-(9-ethyl-9*H*-carbazol-3-yl)-5-methyl-4,7-dihydrotetrazolo[1,5-a]pyrimidine-6-carboxamido)phenyl)sulfonyl)phenyl)-5-methyl-4,7-dihydrotetrazolo[1,5-a]pyrimidine-6-carboxamide (Scheme 1, Product a_15_)


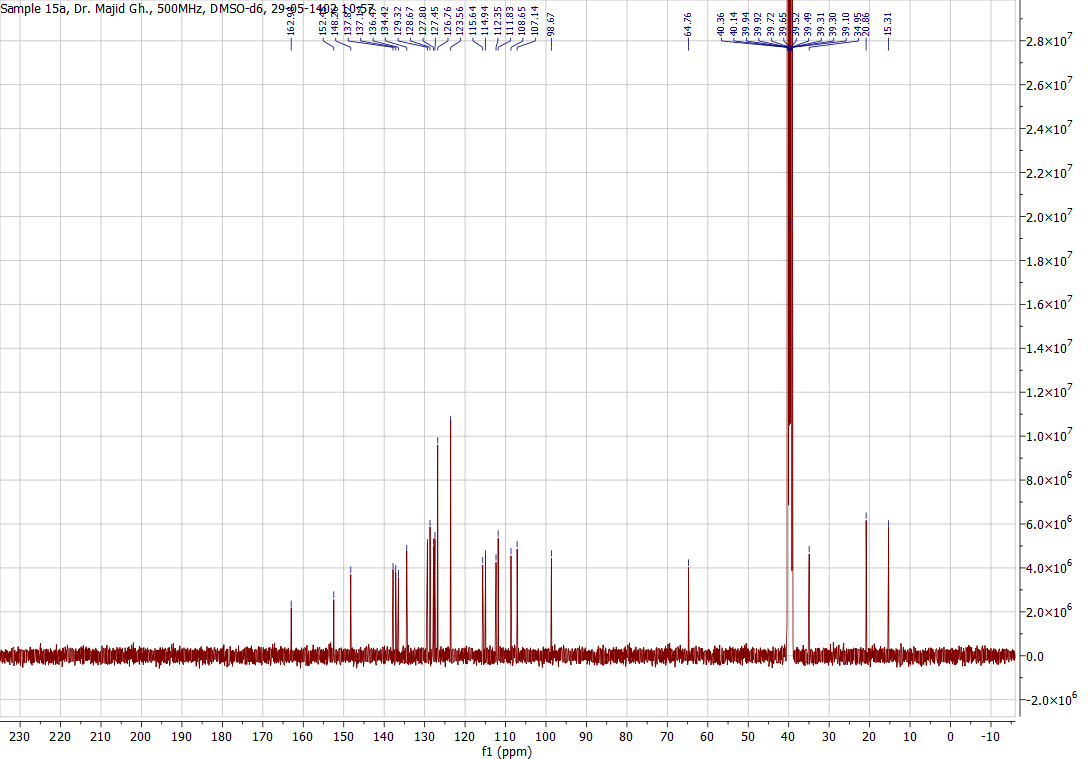


**Supplementary Figure 30:** ^13^C-NMR spectrum of 7-(9-ethyl-9*H*-carbazol-2-yl)-*N*-(4-((4-(7-(9-ethyl-9*H*-carbazol-3-yl)-5-methyl-4,7-dihydrotetrazolo[1,5-a]pyrimidine-6-carboxamido)phenyl)sulfonyl)phenyl)-5-methyl-4,7-dihydrotetrazolo[1,5-a]pyrimidine-6-carboxamide (Scheme 1, Product a_15_)


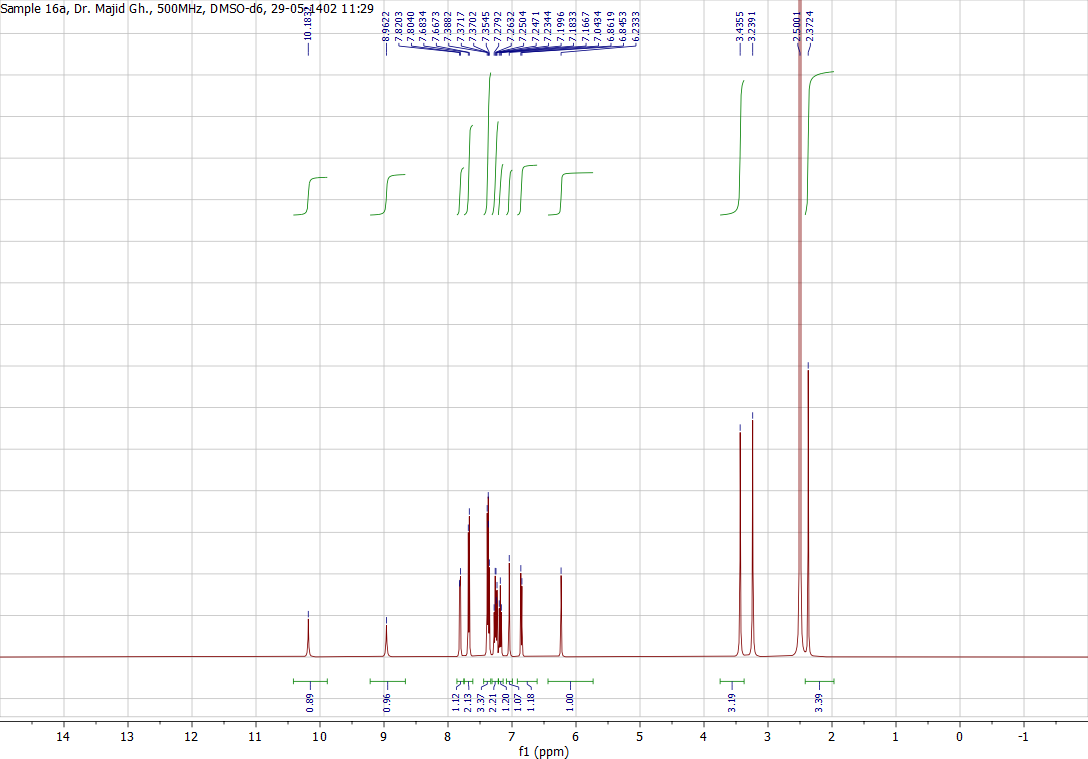


**Supplementary Figure 31:** ^1^H-NMR spectrum of 5-methyl-*N*-(4-((4-(5-methyl-7-(9-methyl-9*H*-carbazol-2-yl)-4,7-dihydrotetrazolo[1,5-a]pyrimidine-6-carboxamido)phenyl)sulfonyl)phenyl)-7-(9-methyl-9*H*-carbazol-3-yl)-4,7-dihydrotetrazolo[1,5-a]pyrimidine-6-carboxamide (Scheme 1, Product a_16_)


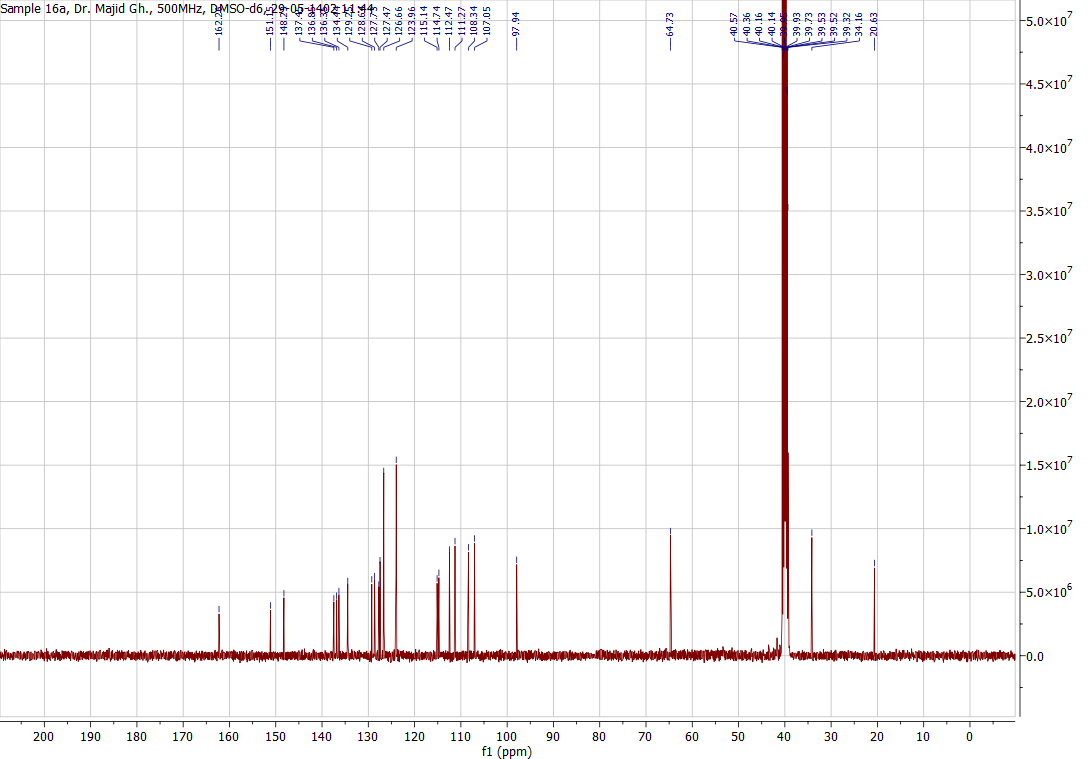


**Supplementary Figure 32:** ^13^C-NMR spectrum of 5-methyl-*N*-(4-((4-(5-methyl-7-(9-methyl-9*H*-carbazol-2-yl)-4,7-dihydrotetrazolo[1,5-a]pyrimidine-6-carboxamido)phenyl)sulfonyl)phenyl)-7-(9-methyl-9*H*-carbazol-3-yl)-4,7-dihydrotetrazolo[1,5-a]pyrimidine-6-carboxamide (Scheme 1, Product a_16_)

1. *Corresponding author.

   *E-mail address:* khalaj_mehdi@yahoo.com (M. Khalaj) and molakhatami@gmail.com (S. M. Khatami) [↑](#footnote-ref-1)
